# Chronic paternal morphine exposure increases sensitivity to morphine-derived pain relief in male progeny

**DOI:** 10.1126/sciadv.abk2425

**Published:** 2022-02-16

**Authors:** Andre B. Toussaint, William Foster, Jessica M. Jones, Samuel Kaufmann, Meghan Wachira, Robert Hughes, Angela R. Bongiovanni, Sydney T. Famularo, Benjamin P. Dunham, Ryan Schwark, Reza Karbalaei, Carmen Dressler, Charlotte C. Bavley, Nathan T. Fried, Mathieu E. Wimmer, Ishmail Abdus-Saboor

**Affiliations:** 1 Department of Psychology, Program in Neuroscience Temple University, Philadelphia, PA, USA.; 2 Zuckerman Mind Brain Behavior Institute and Department of Biological Sciences, Columbia University, New York, NY, USA.; 3 Department of Biology, Rutgers Camden University, Camden, NJ, USA.

## Abstract

Parental history of opioid exposure is seldom considered when prescribing opioids for pain relief. To explore whether parental opioid exposure may affect sensitivity to morphine in offspring, we developed a “rat pain scale” with high-speed imaging, machine learning, and mathematical modeling in a multigenerational model of paternal morphine self-administration. We find that the most commonly used tool to measure mechanical sensitivity in rodents, the von Frey hair, is not painful in rats during baseline conditions. We also find that male progeny of morphine-treated sires had no baseline changes in mechanical pain sensitivity but were more sensitive to the pain-relieving effects of morphine. Using RNA sequencing across pain-relevant brain regions, we identify gene expression changes within the regulator of G protein signaling family of proteins that may underlie this multigenerational phenotype. Together, this rat pain scale revealed that paternal opioid exposure increases sensitivity to morphine’s pain-relieving effects in male offspring.

## Teaser

Prenatal paternal morphine exposure increases morphine sensitivity in progeny.

## INTRODUCTION

Prenatal environmental factors have profound and long-lasting influences on behavior and physiology (*1*–*9*). For example, paternal opioid exposure alters memory, reward processing, anxiety, and aggression in offspring (*7*, *10*–*12*). However, relatively little is known about prenatal morphine’s impact on pain. Previous studies have shown that prenatal exposure to opioids can lead to changes in sensitivity to opioid-derived antinociception (*13*, *14*). However, the regimen and nature of the exposure of the parental generation, consisting of high acute doses to both sires and dams, limited the possible interpretation and translation of these findings to clinical populations. Here, we focused on the paternal lineage because it lays the foundation for better understanding of biological processes underlying the transmission of chronic opioid exposure across generations. This approach circumvents potentially confounding factors such as changes in maternal care, which are known to alter behavioral end points related to affect and pain. Moreover, the work presented here leveraged chronic volitional consumption of morphine that closely mimics the chronic exposure related to chronic pain treatment and/or substance abuse. Together, we combined these highly translational approaches with a novel tool to assess pain in rats to systematically delineate changes in baseline mechanical pain processing and antinociception following acute morphine treatment.

Traditional measurements of pain in rodents rely on relatively low-resolution reflexive paw withdrawal assays that use the withdrawal frequency, latency, or threshold as a behavioral proxy for pain sensitivity (*15*, *16*). However, these measures do not provide an objective assessment of an animal’s internal pain state because they rely on the subjective assignment of stimulus intensity by an experimenter and the readout is the same no matter whether the sensory stimuli are innocuous or noxious (*17*–*20*). Recent advances in machine learning, statistical modeling, and high-speed videography have improved the neuroethology of animal behavior (*21*). Hence, we recently developed “mouse pain scales” that use subsecond behavioral features of the paw withdrawal to more objectively assess pain sensitivity in mice with greater sensitivity than traditional methods (*22*, *23*). Thus, we adapted and validated this strategy to develop a “rat pain scale” to revisit the multigenerational impact of morphine exposure on pain sensitivity using a clinically relevant low-dose self-administration paradigm.

Here, we developed a rat pain scale with both sexes of two outbred strains (Sprague-Dawley and Long-Evans) using high-speed recording of rapid paw and eye kinematics in response to a range of innocuous and noxious mechanical stimuli, demonstrating a sensitive high-resolution method for identifying subtle changes in pain sensitivity. Using this pain scale, we found that von Frey hairs (VFHs), which test mechanical thresholds and are commonly assumed to assess pain sensitivity, were instead causing paw withdrawal movements more similar to those associated with innocuous stimuli. This suggested that VFHs may not be probing pain-specific neurocircuitry. We leveraged the new pain scale to assess morphine antinociception in male offspring of morphine-exposed sires in our recently described multigenerational morphine exposure paradigm (*12*). Last, we used RNA sequencing to delineate changes in expression in brain regions involved in pain including the periaqueductal gray (PAG), ventral tegmental area (VTA), and nucleus accumbens (NAc). Together, this work demonstrates the utility of this newly developed rat pain scale and a potential mechanism for the multigenerational impact of prenatal morphine exposure on sensitivity to morphine’s pain-relieving effects.

## RESULTS

### Constructing a rat pain scale to capture subsecond behavioral responses to evaluate the rat’s internal pain state

**Fig. 1.**
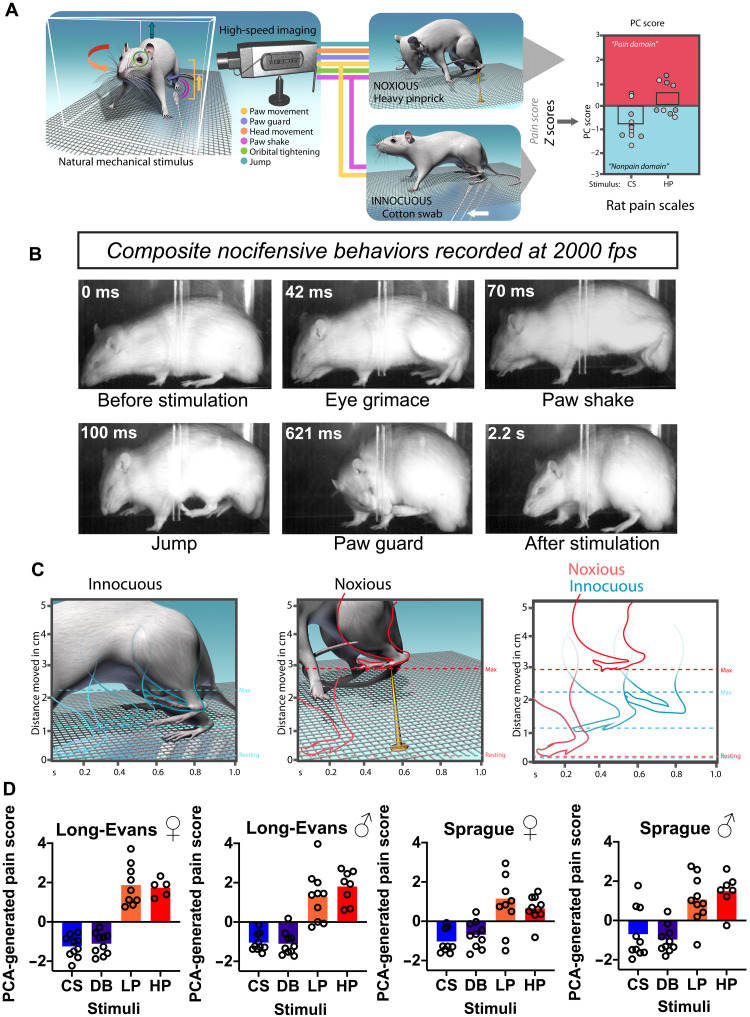
Temporal mapping of affective behaviors, paw kinematic, and principal components analysis–generated pain scores of rats in response to mechanical stimuli. (**A**) Schematic of behavioral procedure and analysis showing lateral placement of high-speed camera in relation to freely behaving rat. Note that the camera lens captures the entire lateral area of the rat. PC, principal component. (**B**) Representative single-frame images taken from high-speed videos of a Sprague-Dawley rat following stimulation with an LP. Each frame captures a distinct nocifensive behavior: eye grimace/orbital tightening (42 ms), paw shake (70 ms), jumping (100 ms), and paw guard (621 ms) fps, frames per second. (**C**) Schematic of hind paw kinematic movements evoked by innocuous (CS and DB) or noxious (LP and HP) stimuli. Paw height (shown on the *y* axis) is the distance from the mesh floor to the highest point following paw stimulation, while paw speed (shown on the *x* axis) is the distance from the initial paw lift to the highest point divided by the time in seconds between the two points. (**D**) PCA-generated pain scores for Long-Evans and Sprague-Dawley female and male rats following stimulation with innocuous (CS and DB) and noxious (LP and HP) stimuli. Negative values are indicative of “nonpain-like,” whereas positive values represents “pain-like” responses.

We used high-speed imaging to capture subsecond behavioral features of the rat paw withdrawal in response to a set of innocuous [cotton swab (CS) and dynamic brush (DB)] and noxious [light pinprick (LP) and heavy pinprick (HP)] natural mechanical stimuli. Using statistical modeling and machine learning, we sought to identify behavioral signatures associated with innocuous versus noxious stimuli for the development of a single-index pain scale (Fig. 1A). A typical response to a noxious stimulus first begins with an eye grimace or orbital tightening (~42 ms), which is consistent with previously reported painful responses in rodents (Fig. 1B) (*24*). Next, the rat raises its hind paw away from the stimulus, holds it at the apex, and then vigorously exhibits a sinusoidal paw shake (~70 ms) with some also jumping in the air (~100 ms). The rat then orients its head toward the stimulus to guard the affected paw (Fig. 1B; ~621 ms; see movie S1, for example, of the four nocifensive behaviors). These four individual behavioral features (orbital tightening, paw shake, jumping, and paw guarding) were combined into one composite nocifensive score and were statistically more common in response to noxious stimuli (HP and LP) versus innocuous stimuli (DB and CS) in males and females of both strains (fig. S1A). To further distinguish paw withdrawals induced by noxious versus innocuous stimuli, we measured subsecond paw kinematics by extracting the speed and height of the first upward paw movement in response to the stimulus (Fig. 1C). We found that the speed and height were both statistically greater in paw withdrawals induced by noxious stimuli (HP and LP) than by innocuous stimuli (DB and CS) in female and males of both strains (fig. S1, B and C).

Nocifensive behaviors and paw kinematics rely on distinct features of the paw withdrawal response to consistently distinguish innocuous from noxious stimuli. Each parameter holds a different dimension of statistical space, differing by unit and value of measurement. To comprehensively analyze and combine these measurements and to normalize each data point with respect to the population mean, we first converted all raw data to normalized *z* scores (table S1). We subsequently combined all *z* scores into a one-dimensional score using principal components analysis (PCA), creating a PCA-generated pain score (PC1 score) that encompassed three behavioral dimensions and provided a threshold separating innocuous from noxious stimuli (fig. S2A). We found that Long-Evans and Sprague-Dawley female and male rats had higher PCA-generated pain scores when stimulated with noxious stimuli (LP and HP) compared to innocuous stimuli (CS and DB) (Fig. 1D, Long-Evans female: *F*(1.836,14.07) = 63.14, *P* < 0.0001; Long-Evans male: *F*(2.155,24.43) = 29.81, *P* < 0.0001; Sprague-Dawley female: *F*(1.544,13.38) = 10.20, *P* = 0.0033; Sprague-Dawley male: *F*(2.001,16.00) = 18.99, *P* < 0.0001). These scores provide a continuous gradation where more positive values are more likely to be produced from noxious stimuli while more negative values are more likely to be produced from innocuous stimuli. In response to HP hind paw stimulation, the PCA-generated pain score for only Long-Evans female rats were significantly higher than female Sprague-Dawley rats, suggesting a strain difference in noxious stimuli in females (Long-Evans females HP versus Sprague-Dawley females HP: *P* = 0.0146; Fig. 1D). Together, this PCA-generated scoring method in rats distinguished innocuous from noxious stimuli for a given trial, and it can be used to map a unique “pain state” in response to mechanical stimuli.

To further measure pain sensation on the basis of behavioral responsivity, we used a machine learning approach to predict the probability of each trial being pain-like, which reliably enable predictions about the pain state of each trial for unscored datasets (fig. S2B). Together, this statistical and machine learning approach provides an objective and highly sensitive method to assess pain in rats.

### VFH filaments do not elicit pain-like responses

**Fig. 2.**
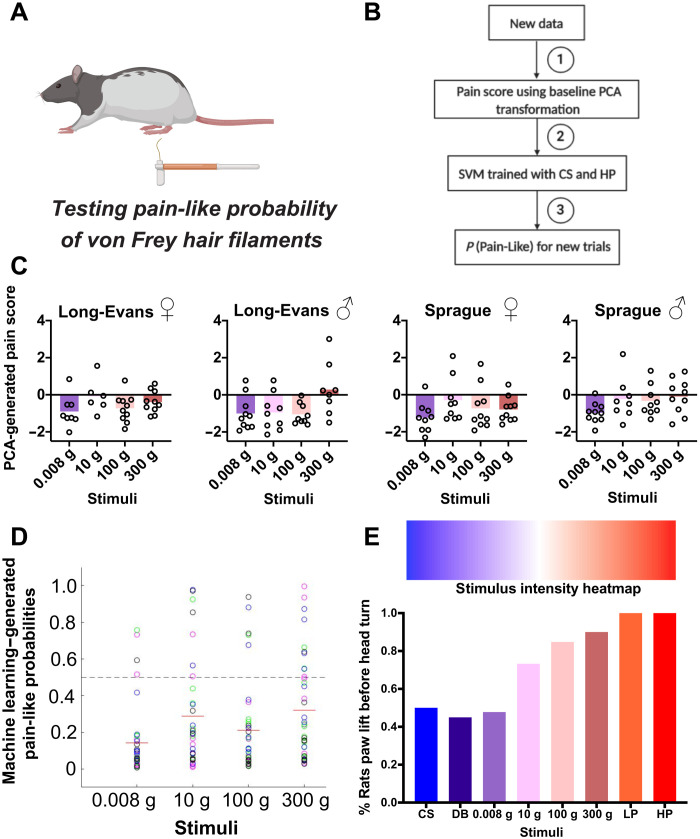
Subsecond temporal mapping of rat behavioral profile in response to VFH filaments. (**A**) Schematic and (**B**) pain scale workflow: (1) PC1 scores obtained using the previous baseline (CS, DB, LP, and HP) PCA transformation, (2) predict on VF trials using the support vector machine (SVM) trained on CS and HP baseline trials, and (3) generate probability pain-like on a trial-by-trial basis for the new data. (**C**) PCA-generated pain scores representing linear transformation of affective and reflexive behavioral features. Negative values are indicative of nonpain-like, whereas positive values are pain-like. (**D**) Machine learning predictions made in Long-Evans and Sprague-Dawley female and male rats of pain-like probabilities of the same VFH filaments. Color key: green, Long-Evans females; magenta, Long-Evans males; black, Sprague-Dawley females; blue, Sprague-Dawley males. (**E**) Stimulus intensity heatmap showing the percentage (*y* axis) of rats that lifted their hind paw before turning to look at stimuli of increasing intensity. *N* = 10 rats of each strain and sex per stimulus.

VFHs are a mainstay in preclinical research, and yet discrepancies in the true nature of the sensation evoked by VFHs persist—evidenced by a lack of consensus for whether a particular filament is noxious or innocuous when testing baseline mechanical thresholds (*25*, *26*). We used four VFHs ranging from the lowest to highest intensity traditionally used in rat (0.008, 10, 100, and 300 g of force; Fig. 2A). Beginning with scoring the affective features that compose the nocifensive composite score, we did not observe any statistically significant difference across any of the VFHs (fig. S3). We next reduced the VFH behavioral measurements into a single “pain scale” dimension using the data transformations derived from the PCA and machine learning approaches described above (Fig. 2B). Corresponding to results obtained with the individual behavioral features (table S2), the PCA-generated pain scores across sex and strain revealed mainly negative values, indicative of nonpain responses (Fig. 2C). For males of each strain, however, the PCA-generated pain scores, mainly of the higher forces, were close to the threshold separating pain from nonpain, without approaching values previously obtained with pinprick stimuli. The complementary machine learning approach across strain and sex revealed that all VFHs have pain-like probabilities below 50%, indicating a low probability of being pain-like (Fig. 2D).

Given this unexpected result to stimulation with VFHs, we analyzed additional subsecond whole-body features in response to VFHs, HP, LP, DB, and CS stimuli. We focused on body orientation as a behavioral readout with the rationale that painful stimuli would result in movement of the paw before orientation of the head toward the stimulus as part of an escape behavior. The greater the stimulus intensity, the greater the proportion of animals that lifted their paw before orienting their head toward to the stimulus [one-way analysis of variance (ANOVA): *F*(1.750,5.251) = 6.728, *P* = 0.0375; Fig. 2E]. Similar to our results with the PCA and machine learning approaches, the VFHs fell between the responses observed with DB and LP (fig. S4). Together, these results demonstrate that rats may perceive VFHs more similar to touch than pain.

### Assessment of morphine-induced analgesia with VFHs

**Fig. 3.**
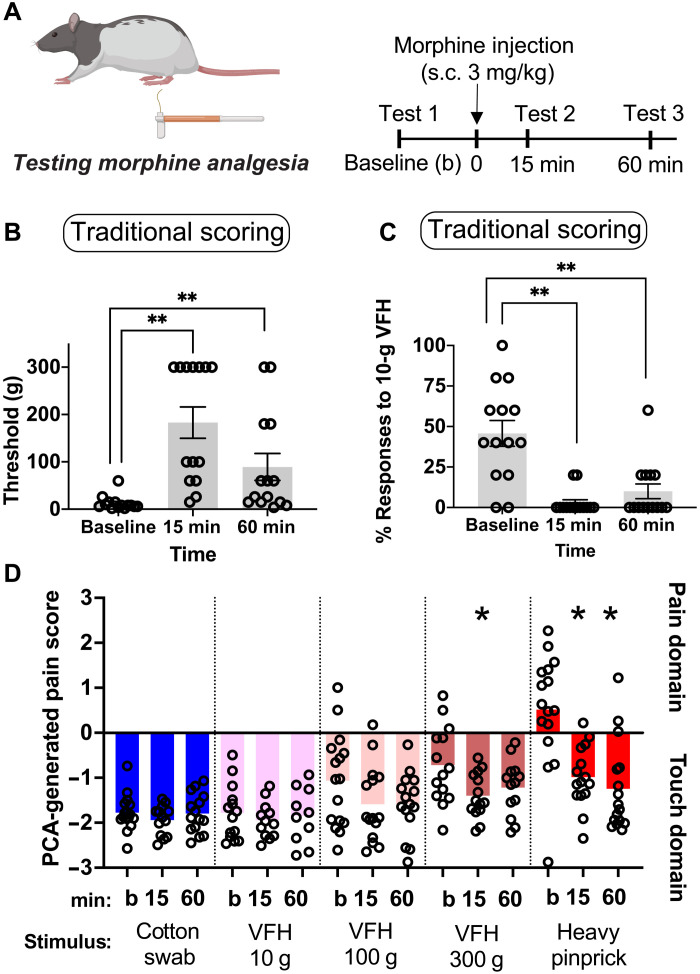
Using traditional scoring and our novel rat pain scale to measure morphine-induced analgesia over time in male rats. (**A**) Schematic and experimental timeline: Long-Evans male rats received baseline (“b”) hind paw mechanical stimulations with VFHs or somatosensory stimuli. Rats then received a subcutaneous injection of morphine (3 mg/kg) and were returned to their home cage. At 15- and 60-min postmorphine injection, rats were returned to the testing chamber to receive hind paw stimulation using the same stimulus and procedure applied at baseline. (**B**) Traditional VFH thresholds after morphine treatment (3 mg/kg). (**C**) Proportion of withdrawal responses at 10-g VFH following morphine treatment. (**D**) Novel rat pain scale PCA-generated pain scores. *P < 0.05; **P < 0.01 compared to baseline measurements.

We then sought to reconcile morphine’s antinociceptive effects identified with traditional VFH scoring that have been well documented with our unexpected observation that VFHs do not appear to be painful. We used both traditional VFH scoring methods and our new rat pain scale to assess the antinociceptive effects of morphine at baseline, 15-min, and 60-min time points (3 mg/kg, subcutaneously; Fig. 3A). A series of VFHs were applied to the plantar surface of the hind paw in ascending order beginning with 1-g force VFH (1, 2, 4, 6, 8, 10, 15, 26, 60, 100, 180, and 300 g). Threshold was defined as the force at which withdrawal responses occurred 40% of the time or more. If the threshold was below 10 g, then five trials were administered to define the percent response at 10 g for each rat. VFH threshold changed after morphine treatment [Long-Evans males: *F*(1.934,25.14) = 17.04, *P* < 0.0001; Fig. 3B]. Sidak post hoc analysis revealed an increase in threshold at 15 and 60 min after morphine injection compared to baseline (15 min, *P* = 0.0003 and 60 min, *P* = 0.0281).

In addition, we generated another commonly reported assessment of pain threshold and pain sensitivity: the percentage of withdrawal responses following stimulation with 10 g of force. Repeated measures ANOVA revealed that the proportion of withdrawal responses at 10-g VFH stimulation decreased following morphine treatment [*F*(1.149,14.94) = 16.12, *P* = 0.0008; Fig. 3C]. Sidak post hoc tests showed that the proportion of withdrawal responses was lower 15 and 60 min after morphine treatment (3 mg/kg) compared to baseline (15 min, *P* = 0.0006; 60 min, *P* = 0.0078). We then assessed the impact of morphine using our novel pain scale with 10-, 100-, and 300-g VFHs to represent forces most commonly considered as painful within the literature. Stimulation with the 10-g VFH and the 100-g VFH elicited nonpain-like PCA scores at baseline, and morphine treatment had no impact on their PCA-generated pain score [10-g VFH: *F*(1.985,18.86) = 0.2979, *P* = 0.7442; 100-g VFH: *F*(1.721,24.09) = 0.3466, *P* = 0.0538; Fig. 3D]. The morphine treatment (3 mg/kg) did produce a statistically significant effect with 300-g VFH force [*F*(1.688,19.41) = 3.931, *P* = 0.0429], although 300 g mapped in the negative “nonpain” domain. Post hoc Sidak tests revealed that the PCA scores in response to 300-g VFH decreased 15 min after morphine treatment compared to baseline (*P* = 0.0297). However, there were no differences in PCA scores comparing 60-min postmorphine injection compared to baseline (*P* = 0.2566). Using HP as a known painful stimulus, we observed that the initial hind paw stimulation generated a positive PCA score, consistent with a pain-like withdrawal reflex. Treatment with morphine had a significant analgesic response at 15 and 60 min after the injection [*F*(1.568,22.73) = 20.33, *P* < 0.001; post hoc Sidak tests: 15 min: *P* = 0.0027; 60 min: *P* < 0.0001 compared to baseline]. Both of these methods would suggest morphine’s potent antinociceptive effects, consistent with the well-characterized changes in response to VFH stimulations after morphine treatment (*27*–*30*).

### Offspring of morphine-exposed sires show increased sensitivity to morphine-induced antinociception

**Fig. 4.**
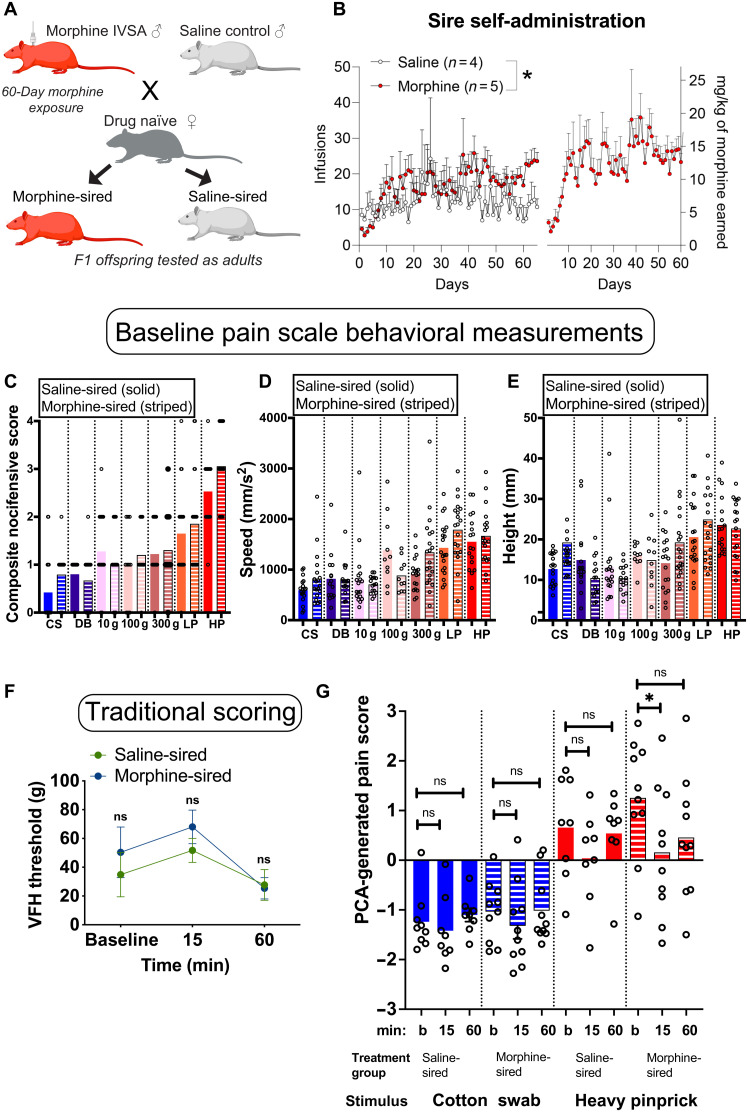
Multigenerational model of paternal morphine exposure used to assess pain in male F1 offspring. (**A**) Experimental paradigm and mating scheme for morphine intravenous self- administration exposure. See Materials and Methods for further details of the model. (**B**) Male Sprague-Dawley rats self-administered morphine for 60 days and throughout breeding with drug-naïve females; controls received saline. Morphine-treated sires earned more infusions than their saline counterparts (left), and the total amount of morphine consumed increased over time (right). (**C**) Composite nocifensive score of F1 offspring following stimulation with the same baseline stimuli. (**D** and **E**) Hind paw speed and height kinematic movements evoked by the same stimuli. (**F**) Traditional scoring measuring VFH threshold does not separate treatment groups over time. (**G**) PCA-generated pain score predictions made in F1 offspring. Negative values are indicative of nonpain-like, whereas positive values pain-like. *P < 0.05. ns, not significant.

With a new mechanical nociception measurement platform created, we next sought to address the question of whether chronic paternal morphine treatment altered morphine-induced analgesia in offspring. Sires self-administered morphine for 60 days, while controls were exposed to the same environment (operant boxes, cues, and levers) but received only saline. Sires were then bred to drug-naïve dams to produce first-generation (F1) offspring. Sires continued to self-administer morphine during the 5-day mating period to avoid withdrawal-related stress as a confounding factor (Fig. 4A). There was an overall difference between saline and morphine consumption, with morphine-treated sires earning more infusions than saline controls [effect of treatment: *F*(1,455) = 37.82, *P* < 0.0001; effect of days: *F*(64,455) = 0.9796, *P* = 0.5244; interaction: *F*(64,455) = 0.6732, *P* = 0.9740; Fig. 4B]. Pups derived from saline-treated or morphine-treated sires were weaned at 21 days of age and group-housed with same sex littermates until behavioral assessments as adults (60 + days of age). Baseline assessments using mechanical stimuli revealed no difference in pain-like responses in male morphine-sired animals compared to saline-sired controls (saline-sired males versus morphine-sired males: CS: *P* = 0.0748; DB: *P* = 0.4929; 10-g VFH: *P* = 0.3095; 100-g VFH: *P* = 0.5480; 300-g VFH: *P* = 0.7157; LP: *P* = 0.5145; HP: *P* = 0.0862; Fig. 4, C to E, and table S3).

Baseline PC scores were assessed before injection of morphine (1 mg/kg) subcutaneously and at the following 15- and 60-min time points (Fig. 4B). We used this low dose of morphine that lies at the threshold of antinociceptive properties to detect any potential increase in sensitivity based on prior work involving prenatal opioid exposure. Using traditional methods, we found that the threshold for pain changed in both saline- and morphine-sired progeny after morphine treatment [Sire: *F*(1,10) = 0.61, *P* = 0.4529; time: *F*(2,20) = 4.934, *P* = 0.0181; interaction: *F*(2,20) = 0.4936, *P* = 0.6177; Fig. 4F]. Post hoc analyses revealed that neither saline-sired rats (*P* = 0.4749 and *P* = 0.8691) nor morphine-sired rats (*P* = 0.4420 and *P* = 0.2103) showed an increase in the VFH threshold 15 or 60 min after morphine injection compared to baseline. These results suggest that paternal morphine exposure did not alter the sensitivity to morphine-derived antinociception. We then used a similar design combined with the pain scale. Following this baseline recording, rats received a subcutaneous injection of low-dose morphine (1 mg/kg) and were returned to their home cage. Rats were tested again with the same stimulus 15 and 60 min after the morphine injection. Behavioral responses were recorded using high-speed videography at all time points. We used the composite nocifensive scores, speed, and height to generate PCA pain scores (table S4). One-way ANOVA revealed that the nature of stimuli influenced the pain score regardless of siring [*F*(17,150) = 6.623, *P* < 0.0001], with pinpricks registering higher scores than innocuous stimuli. Morphine had no impact on the PCA-generated pain score in response to CS stimulation [time: *F*(1,26 = 1.34, *P* = 0.2795; sire: *F*(1,18) = 0.4932, *P* = 0.4915; interaction: *F*(2,21) = 0.4057, *P* = 0.9603] for saline-sired (15 min: *P* = 0.8220; 60 min *P* = 0.8353 compared to baseline; Fig. 4G) or morphine-sired male progeny (15 min: *P* = 0.5365; 60 min: *P* = 0.9899 compared to baseline). For saline-sired offspring, this low dose of morphine did not significantly reduce the PCA-generated pain score in response to HP [time: *F*(1,28 = 5.149, *P* = 0.0139; sire: *F*(1,15) = 0.6263, *P* = 0.4410; interaction: *F*(2,30) = 1.272, *P* = 0.2949; post hoc Sidak tests: 15 min: *P* = 0.2909; 60 min: *P* = 0.9671 compared to baseline]. In sharp contrast, morphine-sired male progeny displayed an antinociceptive response to morphine (1 mg/kg) after stimulation with HP (15 min: *P* = 0.0370; 60 min: *P* = 0.1039 compared to baseline). In particular, the antinociceptive response to low-dose morphine in morphine-sired rats was driven by a decrease in the more affective nocifensive responses and not the reflexive speed and height measurements (fig. S5). Together, our pain scale demonstrates that offspring of morphine-exposed sires are more sensitive to the antinociceptive properties of morphine—a finding that would have been missed with a traditional scoring method.

### Paternal morphine exposure produces region-specific changes in gene expression in the NAc, VTA, and PAG

**Fig. 5.**
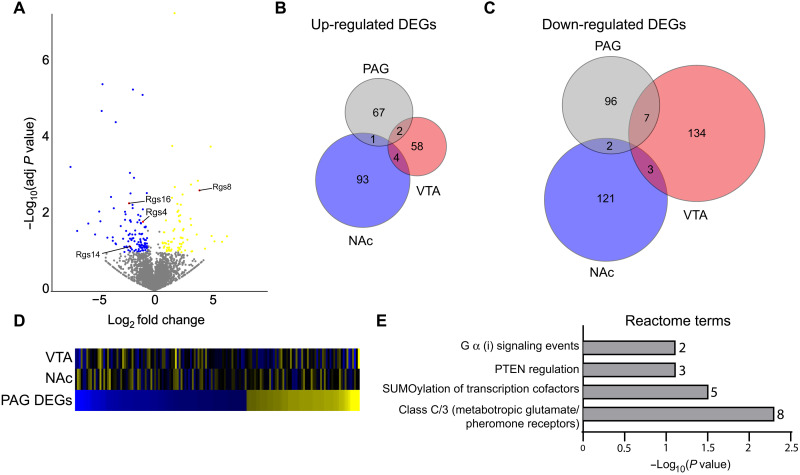
RNA sequencing reveals gene expression changes in the brain associated with the chronic morphine paradigm. (**A**) Volcano plot showing up-regulated (yellow) and down-regulated (blue) genes in morphine-sired versus saline-sired PAG. RGS family genes are labeled and highlighted in red. (**B**) Venn diagram showing the number of up-regulated differentially expressed genes (DEGs) in the PAG versus the VTA and NAc. (**C**) Venn diagram showing the number of down-regulated DEGs in the PAG versus the VTA and NAc. (**D**) Heatmap sorted by fold change of PAG DEGs (bottom) compared to NAc (middle) and VTA (top) DEGs. (**E**) Reactome enrichment terms identified by PAG DEGs. The number of DEGs associated with each term is located to the right of the bar graph. PTEN, Phosphatase and tensin homolog deleted on chromosome 10.

We next examined the potential mechanisms underlying changes in antinociception in male morphine-sired progeny. RNA sequencing was performed on brain regions known to contribute to pain processing and antinociception: the NAc, VTA, and PAG. The results regarding the NAc and VTA will be reported elsewhere, and we compared these findings to the transcriptome of the PAG. We found a total of 175 genes (*x* = 105 down-regulated, 70 up-regulated) differentially expressed in the PAG as a result of paternal morphine exposure (Fig. 5A and table S5). Intriguingly, several genes coding for members of the regulator of G protein signaling (RGS) family of proteins, which have a well-established role in modulating opioid signaling, were affected by morphine siring, with *Rgs4*, *Rgs14*, and *Rgs16* down-regulated and *Rgs8* up-regulated in the PAG of morphine-sired offspring. A history of morphine exposure in the paternal lineage produced distinct transcriptomic alterations in NAc, VTA, and PAG (Fig. 5, B and C), and the expression of genes that were affected in the PAG was largely unaffected in the NAc and VTA of morphine-sired male progeny (Fig. 5D). Enrichment analyses revealed several pathways were affected by paternal morphine exposure including glutamatergic signaling, known to modulate pain in the PAG, and G a I signaling events. Intriguingly, the enrichment of these pathways was largely driven by RGS-related genes (table S6). Overall, these findings provide potential mechanisms by which changes in morphine-derived antinociception is increased in morphine-sired male progeny.

## DISCUSSION

We developed a rat pain scale by recording subsecond behavioral responses to mechanical stimuli and mapping this multidimensional behavioral dataset into a single dimension with PCA and machine learning. We validated this pain scale across several stimuli in both sexes of two outbred rat strains. Unexpectedly, this pain scale identified that the sensation evoked by VFH filaments was largely in the touch and not the painful domain. Last, we used this pain scale to assess the antinociceptive properties of morphine in a multigenerational model of paternal morphine exposure and demonstrated that the offspring produced by morphine-treated sires are hypersensitive to the antinociceptive effects of morphine.

### VFHs do not evoke pain in rats under baseline conditions

VFHs are the most common stimulus used in all of pain research, both in rodents and in the clinic (*31*). Although the traditional readouts from the application of VFHs in rodents (threshold or response frequency) are based simply off detecting a mechanical stimulus, the delivery of VFHs has become synonymous with induction of pain. When we constructed a mouse pain scale using an analogous approach to the work presented here, we noticed that the higher forces of VFHs (relative to mouse size), evoked clear pain-like withdrawals (*22*). However, we find that VFHs as high as 300 g did not evoke pain-like withdrawals in rats. These results demonstrate that caution must be taken when interpreting sensation based simply on the presence of a withdrawal reflex. Rather, the quality of this withdrawal reflex might provide a closer approximation of the animals underlying pain state. Notably, even in humans, maximum-force VFHs do not always induce pain-like sensations, depending on the region of the body stimulated (*32*). Therefore, it is possible that sensory perceptions to stimuli may not be shared across all species, and thus, translating behavioral methodology from one species to another should be treated with caution. Furthermore, when using VFHs to study the neurobiology of sensory systems, one must consider the baseline sensation in a given animal model. Our rat pain scale provides the sensitivity to accomplish this with VFHs, revealing that rat studies relying on VFHs as the primary end point are likely exploring mechanical allodynia and not mechanical hyperalgesia. It is possible that these touch-like paw withdrawals are due to the diameter of the VFH tip, which is blunted, and thus a sharp-tipped VFH may be more appropriate for measuring hyperalgesia with a range of forces. It will be interesting in future directions to determine whether the behavioral responses to VFHs in rats do become more pain-like after induction of various chronic pain models.

Assessment of morphine’s well-reported antinociceptive effects with VFHs also yielded unexpected results. Although morphine clearly decreased the VFH paw withdrawal threshold and frequency, our pain scale found no changes following morphine treatment. Given our finding that VFHs likely do not evoke pain sensation and that morphine has no effect on touch sensation, we conclude that previous studies using VFHs in morphine treatment paradigms at these doses may actually be measuring the reported slight sedative effect on motor responses without perturbing pain-like behaviors (*33*–*36*). Further supporting this interpretation is that our pain scale does indeed confirm morphine’s antinociceptive effects to pinprick stimuli.

### Paternal morphine exposure has long-lasting consequences for morphine-derived antinociception in male progeny

Prenatal opioid exposure has profound consequences for cognition, reward sensitivity, and pain thresholds in the next generation (*37*). Relatively few multigenerational opioid exposure studies have focused on pain sensitivity in the next generation. Oral morphine administration in sires produced male offspring with increased sensitivity to morphine (7 mg/kg) on a hot-plate latency pain test (*38*). Male progeny derived from sires treated with a high dose of morphine (25 mg/kg) acutely had increased sensitivity to the antinociceptive effect of opioids at higher doses (10 and 12 mg/kg of morphine). In sharp contrast, female offspring produced by sires treated with morphine showed similar levels of morphine-derived analgesia (*14*). Chronic morphine exposure of both dams and sires also increased the analgesic properties of a low dose (1.5 mg/kg) of morphine in male progeny in a formalin-based pain assay (*13*). When paternal morphine exposure occurred during adolescence, the impact on nociception in male progeny was more subtle, mildly affecting baseline pain responses (*39*). Overall, it is clear that the nature and duration of parental or paternal morphine exposure is critical in determining the outcomes for the next generation.

The current experiments relied on a highly translational model of opioid exposure using intravenous drug self-administration and an extended regimen that covers the sensitive period of spermatogenesis, which had never been done before. This approach offers a number of advantages, including the ability of sires to titrate the dose of morphine over this long duration and accounts for any development of tolerance over the course of the experiment. By narrowing our focus to the paternal lineage, we laid the groundwork for further investigation into the mechanisms underlying the transmission of the paternal morphine exposure. We have previously shown that maternal behavior is not affected in dams bred to morphine-treated sires, which is a potential caveat in many of the aforementioned studies. Our unique approach combining the novel pain scale with a self-administration–based exposure protocol is an important first step in delineating whether and to what extent these observations may extend to human and clinical populations. Both the paternal exposure and the doses of morphine used to assess pain in progeny represent a substantial advance over previous research. These regimens are akin to doses used for pain management in patients. Moreover, the lower doses of morphine used in the current studies in the exposure model are not confounded by the potentially sedating effects associated with opioid treatments at higher doses (*33*–*36*). By traditional methods to measure pain, the differences in antinociception were missed in this multigenerational paradigm. Hence, the novel pain scale in this study was more sensitive to the effects of chronic paternal morphine exposure on morphine-derived antinociception in F1 progeny. This underscores the robustness of this new rapid approach to measuring mechanical nociception.

To better understand the potential mechanisms underlying increased morphine-derived antinociception in progeny of morphine-exposed sires, we measured changes in gene expression in the PAG region, which has long been known to modulate opioid-related analgesia (*40*–*43*). The PAG had a unique transcriptional signature following paternal morphine exposure when compared to the NAc and the VTAs. Of particular interest, decreased expression of several RGS proteins could partly account for the phenotype reported here. Intracellular RGS proteins act as negative regulators of m-opioid receptor signaling by acting as guanosine triphosphatase–accelerating proteins to turn off signaling of the G a and bγ subunits of heterotrimeric G proteins (*44*, *45*). Consistent with this possibility, genetic manipulations of the R4 family of RGS proteins resulted in enhanced antinociception driven by enhanced opioid signaling in the PAG (*46*, *47*). The expression of two members of the R4 RGS family was suppressed in the PAG of morphine-sired male progeny (RGS4 and RGS16), while one was overexpressed (RGS8). RGS14, which belongs to the R12 subfamily of RGS proteins was also down-regulated by a history of paternal morphine exposure. These findings have laid the groundwork to better understand neurotranscriptomic changes elicited by parental morphine exposure. Overall, these findings have potentially profound implications for pain management of patients raised by parents with a history of opioid exposure.

## MATERIALS AND METHODS

### Animals

Sires and dams (F0 generation) were Sprague-Dawley and Long-Evans rats weighing 250 to 300 g obtained from (Taconic Biosciences, Hudson, NY, USA). Sires were housed individually except for 1 week of pair housing during the mating period. Food and water were available ad libitum, and rats were kept on a 12-hour–12-hour light-dark cycle. Experiments were performed during the light phase. All animal care and experiments were approved by the Institutional Animal Care and Use Committee of the University of Pennsylvania, Columbia University, and Temple University and conducted following the National Institutes of Health guidelines. For establishing the rat pain scale, 20 Sprague-Dawley (10 males and 10 females) and 20 Long-Evans (10 males and 10 females) were used. For multigenerational studies 18 Sprague-Dawley rats (9 males and 9 females) were used to produce a total of 112 F1 pups (males: 28 saline-sired, 32 morphine-sired; females: 22 saline-sired, 30 morphine-sired). Animals were randomly assigned to groups, with two to four rats from a single litter used in the studies as to avoid overrepresentation of any given litter. Whenever possible both males and females were tested, and analyses were conducted separately for each sex. For some of the studies, including the multigenerational drug exposure experiments, only male progeny was included in this report. Data are never collapsed across sex.

### Drugs

Morphine sulfate was a gift from the National Institute on Drug Abuse (NIDA) drug supply or obtained from Spectrum Chemical (Gardena, CA) and dissolved in sterile 0.9% saline.

### Multigenerational morphine exposure model

F1 male and female progeny were produced by breeding sires that self-administered morphine (0.75 mg/kg per infusion; controls received saline) for 60 days to drug-naïve females as previously described (*12*). Sires continued to self-administer morphine during the 5-day breeding period, which began on day 61 of morphine self-administration (see the Supplementary Materials for additional details).

### High-speed videography of stimulus-evoked hind paw withdrawal

Rats were acclimated to a rectangular plexiglass chamber, and stimuli were applied to either the left or right hind paw while recording at 2000 frames per second with a high-speed camera (Photron FastCAM Mini AX 50 170 K-M-32GB - Monochrome 170 K with 32GB memory) and attached lens (Zeiss 2/100 M ZF.2-mount). The apparatus was positioned such that the left paw was more likely to be stimulated, but some trials include stimulation of the right paw because of the position the animal was resting. Pilot data demonstrated no difference in responses between left versus right paws. Paw height/speed and nocifensive behavioral measures were scored offline. Experimenters were blind to treatment group in the context of morphine-sired versus saline-sired animals.

### Machine learning and statistical analysis

We classified paw withdrawal reflexes into “pain” and nonpain categories along a single dimension using a PCA of three measurements obtained from the high-speed imaging data: paw speed, paw height, and composite noficensive score. We could then combine normalized *z* scores for each syllable into a single one-dimensional principal component for every stimulus trial. A classification pipeline consisted of the following steps: (i) the first principal component score (PC1) for each trial was derived from *z* scores of all data; (ii) the principal component 1 scores for training data were used to train a support vector machine (SVM) with radial basis function kernels (kernel coefficient gamma = 1, penalty parameter C = 1); and (iii) for a given trial, the SVM predicts the probability of that the response was pain-like based on that trial’s PC1 score. The data used to generate the PC1 scores and train the SVM for each figure can be seen in table S1.

### RNA sequencing

Brain regions were microdissected and flash-frozen. RNA was extracted and libraries were prepared for sequencing as previously described (*48*), while sequencing reads were aligned and analyzed as previously described (*48*, *49*).

## Supplementary Materials

### This PDF file includes:

Supplementary Methods Figs. S1 to S5 References

## Notes

View/request a protocol for this paper from *Bio-protocol*.

## References

[1] T. Klengel, B. G. Dias, K. J. Ressler, Models of intergenerational and transgenerational transmission of risk for psychopathology in mice. Neuropsychopharmacology 41, 219–231 (2016). 26283147 10.1038/npp.2015.249 PMC4677139

[2] B. G. Dias, K. J. Ressler, Parental olfactory experience influences behavior and neural structure in subsequent generations. Nat. Neurosci. 17, 89–96 (2014). 24292232 10.1038/nn.3594 PMC3923835

[3] Y. Shin Yim, A. Park, J. Berrios, M. Lafourcade, L. M. Pascual, N. Soares, J. Yeon Kim, S. Kim, H. Kim, A. Waisman, D. R. Littman, I. R. Wickersham, M. T. Harnett, J. R. Huh, G. B. Choi, Reversing behavioural abnormalities in mice exposed to maternal inflammation. Nature 549, 482–487 (2017). 28902835 10.1038/nature23909 PMC5796433

[4] R. Kaletsky, R. S. Moore, G. D. Vrla, L. R. Parsons, Z. Gitai, C. T. Murphy, *C. elegans* interprets bacterial non-coding RNAs to learn pathogenic avoidance. Nature 586, 445–451 (2020). 32908307 10.1038/s41586-020-2699-5 PMC8547118

[5] H. Szutorisz, J. A. DiNieri, E. Sweet, G. Egervari, M. Michaelides, J. M. Carter, Y. Ren, M. L. Miller, R. D. Blitzer, Y. L. Hurd, Parental THC exposure leads to compulsive heroin-seeking and altered striatal synaptic plasticity in the subsequent generation. Neuropsychopharmacology 39, 1315–1323 (2014). 24385132 10.1038/npp.2013.352 PMC3988557

[6] N. L. Yohn, M. S. Bartolomei, J. A. Blendy, Multigenerational and transgenerational inheritance of drug exposure: The effects of alcohol, opiates, cocaine, marijuana, and nicotine. Prog. Biophys. Mol. Biol. 118, 21–33 (2015). 25839742 10.1016/j.pbiomolbio.2015.03.002 PMC4459901

[7] L. R. Goldberg, T. J. Gould, Multigenerational and transgenerational effects of paternal exposure to drugs of abuse on behavioral and neural function. Eur. J. Neurosci. 50, 2453–2466 (2019). 29949212 10.1111/ejn.14060 PMC7296771

[8] T. L. Bale, Epigenetic and transgenerational reprogramming of brain development. Nat. Rev. Neurosci. 16, 332–344 (2015). 25921815 10.1038/nrn3818 PMC7064155

[9] B. R. Carone, L. Fauquier, N. Habib, J. M. Shea, C. E. Hart, R. Li, C. Bock, C. Li, H. Gu, P. D. Zamore, A. Meissner, Z. Weng, H. A. Hofmann, N. Friedman, O. J. Rando, Paternally induced transgenerational environmental reprogramming of metabolic gene expression in mammals. Cell 143, 1084–1096 (2010). 21183072 10.1016/j.cell.2010.12.008 PMC3039484

[10] F. M. Vassoler, A. M. Toorie, D. N. Teceno, P. Walia, D. J. Moore, T. D. Patton, E. M. Byrnes, Paternal morphine exposure induces bidirectional effects on cocaine versus opioid self-administration. Neuropharmacology 162, 107852 (2020). 31726075 10.1016/j.neuropharm.2019.107852 PMC8274248

[11] M. Z. Farah Naquiah, R. J. James, S. Suratman, L. S. Lee, M. I. Mohd Hafidz, M. Z. Salleh, L. K. Teh, Transgenerational effects of paternal heroin addiction on anxiety and aggression behavior in male offspring. Behav. Brain Funct. 12, 23 (2016). 27582026 10.1186/s12993-016-0107-y PMC5006377

[12] A. S. Ellis, A. B. Toussaint, M. C. Knouse, A. S. Thomas, A. R. Bongiovanni, H. L. Mayberry, S. Bhakta, K. Peer, D. A. Bangasser, M. E. Wimmer, Paternal morphine self-administration produces object recognition memory deficits in female, but not male offspring. Psychopharmacology 237, 1209–1221 (2020). 31912193 10.1007/s00213-019-05450-6 PMC7124995

[13] G. Ashabi, M. S. Sadat-Shirazi, A. Akbarabadi, N. Vousooghi, Z. Kheiri, H. Toolee, S. Khalifeh, M. R. Zarrindast, Is the nociception mechanism altered in offspring of morphine-abstinent rats? J. Pain 19, 529–541 (2018). 29355609 10.1016/j.jpain.2017.12.268

[14] T. J. Cicero, B. Nock, L. O’Connor, M. Adams, E. R. Meyer, Adverse effects of paternal opiate exposure on offspring development and sensitivity to morphine-induced analgesia. J. Pharmacol. Exp. Ther. 273, 386–392 (1995). 7714793

[15] D. Le Bars, M. Gozariu, S. W. Cadden, Animal models of nociception. Pharmacol. Rev. 53, 597–652 (2001). 11734620

[16] J. S. Mogil, Animal models of pain: Progress and challenges. Nat. Rev. Neurosci. 10, 283–294 (2009). 19259101 10.1038/nrn2606

[17] S. Bourane, K. S. Grossmann, O. Britz, A. Dalet, M. G. Del Barrio, F. J. Stam, L. Garcia-Campmany, S. Koch, M. Goulding, Identification of a spinal circuit for light touch and fine motor control. Cell 160, 503–515 (2015). 25635458 10.1016/j.cell.2015.01.011 PMC4431637

[18] S. S. Ranade, S. H. Woo, A. E. Dubin, R. A. Moshourab, C. Wetzel, M. Petrus, J. Mathur, V. Begay, B. Coste, J. Mainquist, A. J. Wilson, A. G. Francisco, K. Reddy, Z. Z. Qiu, J. N. Wood, G. R. Lewin, A. Patapoutian, Piezo2 is the major transducer of mechanical forces for touch sensation in mice. Nature 516, 121–125 (2014). 25471886 10.1038/nature13980 PMC4380172

[19] B. Duan, L. Cheng, S. Bourane, O. Britz, C. Padilla, L. Garcia-Campmany, M. Krashes, W. Knowlton, T. Velasquez, X. Ren, S. E. Ross, B. B. Lowell, Y. Wang, M. Goulding, Q. Ma, Identification of spinal circuits transmitting and gating mechanical pain. Cell 159, 1417–1432 (2014). 25467445 10.1016/j.cell.2014.11.003 PMC4258511

[20] S. E. Murthy, M. C. Loud, I. Daou, K. L. Marshall, F. Schwaller, J. Kuhnemund, A. G. Francisco, W. T. Keenan, A. E. Dubin, G. R. Lewin, A. Patapoutian, The mechanosensitive ion channel Piezo2 mediates sensitivity to mechanical pain in mice. Sci. Transl. Med. 10, eaat9897 (2018). 30305457 10.1126/scitranslmed.aat9897 PMC6709986

[21] N. T. Fried, A. Chamessian, M. J. Zylka, I. Abdus-Saboor, Improving pain assessment in mice and rats with advanced videography and computational approaches. Pain 161, 1420–1424 (2020). 32102021 10.1097/j.pain.0000000000001843 PMC7302333

[22] I. Abdus-Saboor, N. T. Fried, M. Lay, J. Burdge, K. Swanson, R. Fischer, J. Jones, P. Dong, W. Cai, X. Guo, Y. X. Tao, J. Bethea, M. Ma, X. Dong, L. Ding, W. Luo, Development of a mouse pain scale using sub-second behavioral mapping and statistical modeling. Cell Rep. 28, 1623–1634.e4 (2019). 31390574 10.1016/j.celrep.2019.07.017 PMC6724534

[23] J. M. Jones, W. Foster, C. R. Twomey, J. Burdge, O. M. Ahmed, T. D. Pereira, J. A. Wojick, G. Corder, J. B. Plotkin, I. Abdus-Saboor, A machine-vision approach for automated pain measurement at millisecond timescales. eLife 9, e57258 (2020). 32758355 10.7554/eLife.57258 PMC7434442

[24] D. J. Langford, A. L. Bailey, M. L. Chanda, S. E. Clarke, T. E. Drummond, S. Echols, S. Glick, J. Ingrao, T. Klassen-Ross, M. L. Lacroix-Fralish, L. Matsumiya, R. E. Sorge, S. G. Sotocinal, J. M. Tabaka, D. Wong, A. M. van den Maagdenberg, M. D. Ferrari, K. D. Craig, J. S. Mogil, Coding of facial expressions of pain in the laboratory mouse. Nat. Methods 7, 447–449 (2010). 20453868 10.1038/nmeth.1455

[25] A. Francois, N. Schuetter, S. Laffray, J. Sanguesa, A. Pizzoccaro, S. Dubel, A. Mantilleri, J. Nargeot, J. Noel, J. N. Wood, A. Moqrich, O. Pongs, E. Bourinet, The low-threshold calcium channel Cav3.2 determines low-threshold mechanoreceptor function. Cell Rep. 10, 370–382 (2015). 25600872 10.1016/j.celrep.2014.12.042

[26] S. H. Woo, S. Ranade, A. D. Weyer, A. E. Dubin, Y. Baba, Z. Qiu, M. Petrus, T. Miyamoto, K. Reddy, E. A. Lumpkin, C. L. Stucky, A. Patapoutian, Piezo2 is required for Merkel-cell mechanotransduction. Nature 509, 622–626 (2014). 24717433 10.1038/nature13251 PMC4039622

[27] J. Mika, M. Osikowicz, W. Makuch, B. Przewlocka, Minocycline and pentoxifylline attenuate allodynia and hyperalgesia and potentiate the effects of morphine in rat and mouse models of neuropathic pain. Eur. J. Pharmacol. 560, 142–149 (2007). 17307159 10.1016/j.ejphar.2007.01.013

[28] D. G. Williams, A. Dickenson, M. Fitzgerald, R. F. Howard, Developmental regulation of codeine analgesia in the rat. Anesthesiology 100, 92–97 (2004). 14695729 10.1097/00000542-200401000-00017

[29] K. Popiolek-Barczyk, D. Lazewska, G. Latacz, A. Olejarz, W. Makuch, H. Stark, K. Kiec-Kononowicz, J. Mika, Antinociceptive effects of novel histamine H3 and H4 receptor antagonists and their influence on morphine analgesia of neuropathic pain in the mouse. Br. J. Pharmacol. 175, 2897–2910 (2018). 29486058 10.1111/bph.14185 PMC6016676

[30] H. L. Bu, Y. Z. Xia, P. M. Liu, H. M. Guo, C. Yuan, X. C. Fan, C. Huang, Y. Y. Wen, C. L. Kong, T. Wang, L. T. Ma, X. X. Li, H. W. Zhang, L. R. Zhang, M. Y. Ma, Y. Q. Ai, W. Zhang, The roles of chemokine CXCL13 in the development of bone cancer pain and the regulation of morphine analgesia in rats. Neuroscience 406, 62–72 (2019). 30826523 10.1016/j.neuroscience.2019.02.025

[31] M. Barrot, Tests and models of nociception and pain in rodents. Neuroscience 211, 39–50 (2012). 22244975 10.1016/j.neuroscience.2011.12.041

[32] R. Schmidt, M. Schmelz, M. Ringkamp, H. O. Handwerker, H. E. Torebjork, Innervation territories of mechanically activated C nociceptor units in human skin. J. Neurophysiol. 78, 2641–2648 (1997). 9356413 10.1152/jn.1997.78.5.2641

[33] I. Kissin, P. T. Brown, E. L. Bradley Jr., Sedative and hypnotic midazolam-morphine interactions in rats. Anesth. Analg. 71, 137–143 (1990). 2375515 10.1213/00000539-199008000-00005

[34] F. V. Abbott, E. R. Guy, Effects of morphine, pentobarbital and amphetamine on formalin-induced behaviours in infant rats: Sedation versus specific suppression of pain. Pain 62, 303–312 (1995). 8657430 10.1016/0304-3959(94)00277-L

[35] R. Fog, Behavioural effects in rats of morphine and amphetamine and of a combination of the two drugs. Psychopharmacologia 16, 305–312 (1970). 5461437 10.1007/BF00404736

[36] S. Toyama, N. Shimoyama, Y. Tagaito, H. Nagase, T. Saitoh, M. Yanagisawa, M. Shimoyama, Nonpeptide orexin-2 receptor agonist attenuates morphine-induced sedative effects in rats. Anesthesiology 128, 992–1003 (2018). 29521652 10.1097/ALN.0000000000002161

[37] F. M. Vassoler, M. E. Wimmer, Consequences of parental opioid exposure on neurophysiology, behavior, and health in the next generations. Cold Spring Harb. Perspect. Med. 11, a040436 (2021). 32601130 10.1101/cshperspect.a040436 PMC8485740

[38] P. S. Eriksson, L. Ronnback, E. Hansson, Do persistent morphine effects involve interactions with the genome? Drug Alcohol Depend. 24, 39–43 (1989). 2758973 10.1016/0376-8716(89)90006-9

[39] N. Pachenari, H. Azizi, E. Ghasemi, M. Azadi, S. Semnanian, Exposure to opiates in male adolescent rats alters pain perception in the male offspring. Behav. Pharmacol. 29, 255–260 (2018). 29543652 10.1097/FBP.0000000000000388

[40] C. B. Pert, S. H. Snyder, Opiate receptor: Demonstration in nervous tissue. Science 179, 1011–1014 (1973). 4687585 10.1126/science.179.4077.1011

[41] H. Sims-Williams, J. C. Matthews, P. S. Talbot, S. Love-Jones, J. C. Brooks, N. K. Patel, A. E. Pickering, Deep brain stimulation of the periaqueductal gray releases endogenous opioids in humans. NeuroImage 146, 833–842 (2017). 27554530 10.1016/j.neuroimage.2016.08.038 PMC5312788

[42] C. Li, J. A. Sugam, E. G. Lowery-Gionta, Z. A. McElligott, N. M. McCall, A. J. Lopez, J. M. McKlveen, K. E. Pleil, T. L. Kash, Mu opioid receptor modulation of dopamine neurons in the periaqueductal gray/dorsal raphe: A role in regulation of pain. Neuropsychopharmacology 41, 2122–2132 (2016). 26792442 10.1038/npp.2016.12 PMC4908643

[43] D. J. Mayer, T. L. Wolfle, H. Akil, B. Carder, J. C. Liebeskind, Analgesia from electrical stimulation in the brainstem of the rat. Science 174, 1351–1354 (1971). 5167502 10.1126/science.174.4016.1351

[44] S. Hollinger, J. R. Hepler, Cellular regulation of RGS proteins: Modulators and integrators of G protein signaling. Pharmacol. Rev. 54, 527–559 (2002). 12223533 10.1124/pr.54.3.527

[45] N. B. Senese, R. Kandasamy, K. E. Kochan, J. R. Traynor, Regulator of G-protein signaling (RGS) protein modulation of opioid receptor signaling as a potential target for pain management. Front. Mol. Neurosci. 13, 5 (2020). 32038168 10.3389/fnmol.2020.00005 PMC6992652

[46] J. T. Lamberts, E. M. Jutkiewicz, R. M. Mortensen, J. R. Traynor, mu-Opioid receptor coupling to Gα(o) plays an important role in opioid antinociception. Neuropsychopharmacology 36, 2041–2053 (2011). 21654736 10.1038/npp.2011.91 PMC3158321

[47] J. T. Lamberts, J. R. Traynor, Opioid receptor interacting proteins and the control of opioid signaling. Curr. Pharm. Des. 19, 7333–7347 (2013). 23448476 10.2174/138161281942140105160625 PMC6707067

[48] E. O. Sanchez, C. C. Bavley, A. U. Deutschmann, R. Carpenter, D. R. Peterson, R. Karbalaei, J. Flowers II, C. M. Rogers, M. G. Langrehr, C. S. Ardekani, S. T. Famularo, A. R. Bongiovanni, M. C. Knouse, S. B. Floresco, L. A. Briand, M. E. Wimmer, D. A. Bangasser, Early life adversity promotes resilience to opioid addiction-related phenotypes in male rats and sex-specific transcriptional changes. Proc. Natl. Acad. Sci. 118, e2020173118 (2021). 33593913 10.1073/pnas.2020173118 PMC7923376

[49] S. M. E. Sahraeian, M. Mohiyuddin, R. Sebra, H. Tilgner, P. T. Afshar, K. F. Au, N. B. Asadi, M. B. Gerstein, W. H. Wong, M. P. Snyder, E. Schadt, H. Y. K. Lam, Gaining comprehensive biological insight into the transcriptome by performing a broad-spectrum RNA-seq analysis. Nat. Commun. 8, 59 (2017). 28680106 10.1038/s41467-017-00050-4 PMC5498581

[50] L. Cui, X. Miao, L. Liang, I. Abdus-Saboor, W. Olson, M. S. Fleming, M. Ma, Y. X. Tao, W. Luo, Identification of early RET+ deep dorsal spinal cord interneurons in gating pain. Neuron 91, 1137–1153 (2016). 27545714 10.1016/j.neuron.2016.07.038 PMC5017914

[51] S. Andrews, *FastQC: a quality control tool for high throughput sequence data* (Babraham Bioinformatics, Babraham Institute, Cambridge, United Kingdom, 2010).

[52] A. M. Bolger, M. Lohse, B. J. B. Usadel, Trimmomatic: A flexible trimmer for Illumina sequence data. Bioinformatics 30, 2114–2120 (2014). 24695404 10.1093/bioinformatics/btu170 PMC4103590

[53] J. Sirén, N. Välimäki, V. Mäkinen, HISAT2-fast and sensitive alignment against general human population. IEEE/ACM Trans. Comput. Biol. Bioninformatics 11, 375–388 (2014). 10.1109/TCBB.2013.2297101 26355784

[54] M. Pertea, G. M. Pertea, C. M. Antonescu, T.-C. Chang, J. T. Mendell, S. L. J. N. b. Salzberg, StringTie enables improved reconstruction of a transcriptome from RNA-seq reads. Nat. Biotechnol. 33, 290 (2015). 25690850 10.1038/nbt.3122 PMC4643835

[55] M. I. Love, W. Huber, S. Anders, Moderated estimation of fold change and dispersion for RNA-seq data with DESeq2. Genome Biol. 15, 550 (2014). 25516281 10.1186/s13059-014-0550-8 PMC4302049

[56] R Core Team, *R: A Language and Environment for Statistical Computing* (R Foundation for Statistical Computing, 2013).

[57] S. Heinz, C. Benner, N. Spann, E. Bertolino, Y. C. Lin, P. Laslo, J. X. Cheng, C. Murre, H. Singh, C. K. Glass, Simple combinations of lineage-determining transcription factors prime cis-regulatory elements required for macrophage and B cell identities. Mol. Cell 38, 576–589 (2010). 20513432 10.1016/j.molcel.2010.05.004 PMC2898526

[58] G. Yu, Q.-Y. He, ReactomePA: An R/Bioconductor package for reactome pathway analysis and visualization. Mol. BioSyst. 12, 477–479 (2016). 26661513 10.1039/c5mb00663e

[59] S. B. Plaisier, R. Taschereau, J. A. Wong, T. G. Graeber, Rank–rank hypergeometric overlap: Identification of statistically significant overlap between gene-expression signatures. Nucleic Acids Res. 38, e169 (2010). 20660011 10.1093/nar/gkq636 PMC2943622

[60] K. M. Cahill, Z. Huo, G. C. Tseng, R. W. Logan, M. L. Seney, Improved identification of concordant and discordant gene expression signatures using an updated rank-rank hypergeometric overlap approach. Sci. Rep. 8, 9588 (2018). 29942049 10.1038/s41598-018-27903-2 PMC6018631

