# Supplementary material for: Chronic paternal morphine exposure increases sensitivity to morphine-derived pain relief in male progeny
Source: Sci Adv. 2022 Feb 16;8(7):eabk2425. doi: 10.1126/sciadv.abk2425 (PMC8849295; doi:10.1126/sciadv.abk2425)
Supplement: Supplementary file 1 — Supplementary Methods Figs. S1 to S5 References [file sciadv.abk2425_sm.pdf]

Science Advances
AAAS

Supplementary Materials for

Chronic paternal morphine exposure increases sensitivity to morphine-derived pain relief in male progeny

Andre B. Toussaint, William Foster, Jessica M. Jones, Samuel Kaufmann, Meghan Wachira, Robert Hughes, Angela R. Bongiovanni, Sydney T. Famularo, Benjamin P. Dunham, Ryan Schwark, Reza Karbalaei, Carmen Dressler, Charlotte C. Bavley, Nathan T. Fried, Mathieu E. Wimmer, Ishmail Abdus-Saboor*

*Corresponding author. Email: ia2458@columbia.edu

Published 16 February 2022, *Sci. Adv.* **8**, eabk2425 (2022)
DOI: 10.1126/sciadv.abk2425

The PDF file includes:

Supplementary Methods
Figs. S1 to S5
Legend for movie S1
Legends for tables S1 to S6
References

Other Supplementary Material for this manuscript includes the following:

Movie S1
Tables S1 to S6

Supplementary Methods

Multigenerational morphine exposure model

Adult male Sprague Dawley rats were anesthetized using an i.p. injection of a ketamine/xylazine cocktail (80 and 12 mg kg^{-1}, respectively). An indwelling silastic catheter (Instech Laboratories, Inc. Plymouth Meeting, PA) was fed into the right jugular vein, sutured in place, and mounted on the shoulder blade using a back-mount platform. Catheters were flushed daily with timentin (0.93 mg ml^{-1}), dissolved in heparinized saline and sealed using metal caps, when not in use. Following catheterization surgery, rats were allowed to recover for 1 week then were placed in operant chambers to lever press for 3h daily access of infusions of morphine (0.75 mg/kg/infusion over 5 s) for sixty continuous days; control animals underwent the same catheterization surgeries and self-administration protocol but only had access to saline and were never exposed to morphine. Following chronic morphine self-administration, naïve female rats were placed in a cage with each male rat. Paternal stress has been shown to have long lasting consequences for offspring; therefore, sires continued to self-administer during the 5-day mating period to avoid withdrawal-related stress as a confounding factor (Morgan and Bale 2011, 2013, 2015). Sires were then removed and dams reared first-generation F1 progeny independently until post-natal day (PND) 21, at which point pups were weaned and group housed with littermates. Male and female offspring were pair-housed with same sex littermates upon weaning and remained pair-housed throughout behavioral testing. F1 behavioral testing was conducted when offspring were 2–6 months old. One to two animals from each litter were randomly selected for behavioral test, such that no litter was over-represented in any particular experiment.

Drugs

Morphine sulfate was a gift from the NIDA drug supply or obtained from Spectrum Chemical (Gardena, CA) and dissolved in sterile 0.9% saline.

Mechanically evoked hind paw somatosensory paradigm and high speed videography

Rats behavior was recorded at 2000 frames per seconds. The camera was placed at a ~45° angle at ~1-2 feet away from the Plexiglas holding chambers on a tripod with geared head for Photron AX 50. CMVision IP65 infrared lights that rats cannot detect were used to adequately illuminate the paw for subsequent scoring. Data collected on a Dell laptop computer with Photron FastCAM Analysis software.

Rats were acclimated to a rectangular plexiglas chamber where they could move freely but could not stand straight up. We delivered selected mechanical stimuli to the hind paw when rats were calm, still, and all four paws were in contact with the raised mesh platform. Rats habituated to the testing chambers before preforming behavioral tests. For the baseline experiments (CS, DB, LP, and HP) we tested the same number (10) of male and female Sprague Dawley and Long Evans rats, for a total of 40. We applied stimuli to the hind paw of each rat through the mesh floor. Cotton swab tests consisted of gentle contact between the cotton Q-tip and the hind paw of the mouse. Dynamic brush tests were performed by wiping a concealer makeup brush (e.l.f.™, purchased at the CVS) across the hind paw from back to front. We preformed light pinprick tests by gently touching a pin (Austerlitz Insect Pins®) to the hind paw of the mouse. We withdrew pins after achieving contact. We preformed heavy pinprick tests by sharply pressing this pin into the paw, pushing upward with force. The pin was withdrawn as soon as approximately 1/3 of the pins length had passed through the mesh. For application of von Frey hairs (VFHs, Stoelting Company, 58011), we used four different forces: 0.08 g, 10 g, 100 g, and 300 g. These data related to establishing the novel pain scale are shown in Figure 2. As previously described, we

directed each VFH towards the center of the plantar paw and pressed upward until the filament bent [50]. For the four natural stimuli and VFHs, a non-responsive animal did not respond within 2 s of stimulus delivery. For traditional measures using VFH (Figure 3), animals were placed in the same plexiglass enclosure and paw responses were scored as either withdrawn or not (binary outcome). Animals were stimulated 5 times with each of the following VFH: 1g, 2g, 4g, 6g, 8g, 10g, 15g, 26g, 60g, 100g, 180g, 300g. When animals withdrew their paw 2 or more times (40%) at a given force, the experiment stopped and the threshold was reported at that VFH force. If the threshold was below 10g, one more round of stimulation at 10g was performed to establish the percentage of responses at 10g. The purpose of that experiment was to collect data to assess another commonly reported measurement of pain in the scientific literature: proportion of responses at 10g of force. For experiments testing the antinociceptive properties of morphine using VFH stimulations and the novel pain scale, we used 10g, 100g and 300g of force. Each stimulus was measured on a different day and the order in which stimuli were presented was counterbalanced across animals. All experiments were carried out by an experimenter blind to experimental group.

Scoring hind paw withdrawal movement features

We extracted paw height and paw speed from the high-speed videos and processed with Photron FastCAM software. We scored paw height in centimeters as the distance from the mesh floor to the highest point following paw stimulation. We calculated paw speed as the distance, in centimeters, from initial paw lift to the highest point, divided by the time in seconds between the two points. The composite nocifensive score is a composite of four individual behavior features: orbital tightening, paw shake, paw guard, and jumping. For example, if a given animal displayed 1/4 of those features it would receive a composite nocifensive score of 1. We scored orbital

tightening when the eyes went from fully open to partially or fully closed following stimulus application. We defined paw shaking as high frequency paw flinching. We defined jumping as three or more paws off the mesh floor at the same time following a stimulus application. Lastly, we defined paw guard as any abnormal orientation, or placement, of the paw back during the descent of the withdrawal following stimulus application. We were not blind to the strain when scoring behaviors of wild-type rats, as Long Evans have white coats with a black hood color, while Sprague Dawleys are white-coated. However, we are blind to the stimulus type and VFH forces.

Timeseries experimental paradigm

Following 2-minutes of habituation inside of the testing chamber, naïve adult male rats received mechanical stimulation to their hindpaw for approximately 1-2 seconds or until an apparent behavioral response was elicited with 1 of 3 stimuli: cotton swab, light pinprick or heavy pinprick. A different stimulus was used for different groups of rats during the 3-day experiment, with the initial stimulation serving as a baseline measure of pain sensitivity. Rats were then immediately injected with morphine (either 1mg/kg or 3mg/kg subcutaneously) and returned to their home cage. At 15-min and 60 min after the morphine injection, rats were returned to the testing chamber to receive hindpaw stimulation from the same stimulus. Behavioral responses were recorded using high-speed videography at baseline, 15min, and 60min post-injection.

RNA sequencing:

To have an overview of samples and RNA-seq reads, the quality control (QC) step was done using Fastqc software (version 0.11.8) [51]. Based on the Fastqc software report, the Trimmomatic (version 0.39) software [52] was applied to remove non-paired reads and identified adaptors. For RNA-seq analysis, the suggested pipeline by Sahraeian et al. was used [49] as

previously reported [48]. Briefly speaking, the reads were mapped on rn6 genome assembly using the Hisat2 [53] as the alignment step, the assembled by StringTie [54]. The differentially expressed genes (DEGs) were selected by applying Deseq2 library [55] in R (version 4.0.1) [56]. The conditions to define DEGs were a 50% change in the expression ($|\log 2 \text{ Fold change}| > 0.58$) of genes and an adjusted p-value < 0.1. R statistical software (version 4.0.3) was also used for downstream analysis and visualization of the RNA-seq analysis output, including, but not limited to drawing heatmaps and Venn diagrams.

To find the DEGs' transcription factors, HOMER [57] was used to identify potential transcription factors responsible for DEGs. The applied criteria were − 2000 to + 1000 bp of the transcriptional start site and a length of 8–12 bp for TFs.

Pathways (from KEGG) and gene ontology terms (biological process, molecular function and cell compartment) by adjusted p-value < 0.1 were selected for further analysis. Also, in the case of REACTOME as another source of enrichment analysis, the ReactomePA package in R was used [58]. To compare samples' gene expression, agnostically, the Rank–Rank Hypergeometric Overlap (RRHO) analysis was used. This algorithm steps within two gene lists ranked by the p-value of differential expression observed in two experiments and estimating the number of overlapping genes. Subsequently, a heatmap is created that shows the strength and pattern of correlation between two expression profiles[59]. This analysis was done using the RRHO2 library in R [60] to compare gene expression between Nac, VTA and PAG for overlap in up-and downregulated DEGs, respectively. This research includes calculations carried out on Temple University's HPC resources and thus was supported in part by the National Science Foundation through major research instrumentation grant number 1625061 and by the US Army Research Laboratory under contract number W911NF-16-2-0189

Statistical differences in composite nocifensive score, paw speed and paw height of rats in responses to innocuous (CS and DB) and noxious stimuli (LP and HP)

Hind paw stimulation with CS or DB produced significantly lower composite nocifensive scores than LP or HP across both sex and strain (**Supplemental Figure 1A**; Long Evans Female: $F(2.103, 23.13) = 22.06$, $p<0.0001$; Long Evans Male: $F(1.947, 16.88) = 27.37$, $p<0.0001$; Sprague Dawley Female: $F(1.724, 20.11) = 4.969$, $p=0.0213$; Sprague Dawley Male: $F(2.208, 17.67) = 14.47$, $p<0.0001$). Interestingly, when stimulated with HP, Long Evans male rats displayed higher composite nocifensive scores than Sprague female and male rats that were stimulated with either LP or HP (Long Evans Males HP vs. Sprague Dawley Females LP: p= 0.0342; Long Evans Males HP vs. Sprague Dawley Females HP: p= 0.0365; Long Evans Males HP vs. Sprague Dawley Males LP: p= 0.0342). Speed of the hind paw was higher in response to LP and HP compared to innocuous stimuli across strains and sexes (**Supplemental Figure 1B ;** Long Evans Female: $F(2.223, 23.71) = 48.24$, $p<0.0001$; Long Evans Male: $F(1.755, 19.89) = 25.84$, $p<0.0001$; Sprague Dawley Female: $F(1.995, 17.29) = 15.04$, $p=0.0002$; Sprague Dawley Male: $F(1.611, 18.26) = 9.737$, $p=0.0022$). Although latency to withdraw their paw increased with stimulus intensity, no statistical difference was revealed for sex or strain in response to stimulation with a painful LP or HP (Sex and Strain: LP and HP: $F(4.036, 36.90) = 1.454$, $p = 0.2358$). Maximum height of the hind paw was higher in response to LP and HP compared to innocuous stimuli for all strains and sexes (**Supplemental Figure 1C;** Long Evans Female: $F(1.958, 15.01) = 18.28$, $p<0.0001$; Long Evans Male: $F(1.866, 21.14) = 6.786$, $p=0.0061$; Sprague Dawley Female: $F(1.712, 14.84) = 3.922$, $p=0.0483$; Sprague Dawley Male: $F(2.070, 17.25) = 5.805$, $p=0.0112$). Paw lift height increased in a stepwise manner with the application of each stimulus to the rat's hind paw but no significant difference in sex or strain was found in

response to application of the painful LP compared to HP (Sex and Strain: LP and HP: $F(2.927, 22.99) = 0.8906$, $p = 0.4586$).

Toussaint et. al. Supplemental Figure 1

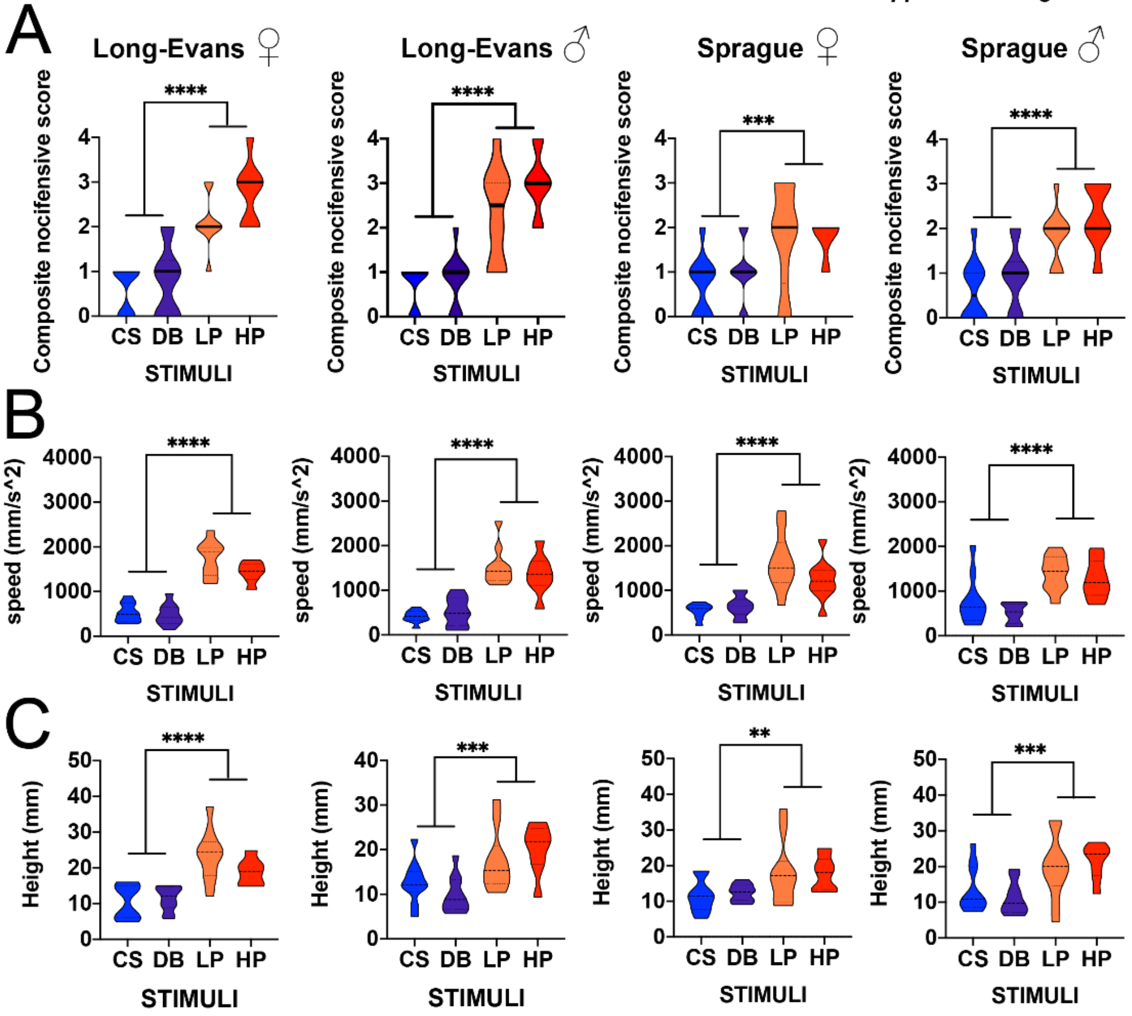

Fig. S2.

Supplemental Figure 1 . **Sub-second temporal mapping of rat paw kinematics in response to mechanical stimuli.** (A) Composite nocifensive score of Long Evans and Sprague Dawley female and male rats (40 rats in total; 10 per group) following stimulation with either a cotton swab (CS), dynamic brush (DB), light pinprick (LP), or heavy pinprick (HP). The score is a composite measurement of eye grimace/orbital tightening, paw shake, jumping, and paw guard. (B and C) The maximum paw height and speed, respectively of the first paw raise of the stimulated paw in Long Evans and Sprague Dawley female and male rats. Truncated violin plots show the distribution density of the composite scores, with the solid horizontal top and bottom lines representing the minimum and maximum values, respectively; dashed horizontal line showing the median value; and dotted top and bottom horizontal lines representing the cutoff for the top 75% and bottom 25% percentile of values, respectively. *** p< 0.0001 and ** p< 0.05.

Linear transformation and machine learning estimates the pain-probability of a given response on a trial-by-trial basis

Machine learning approaches allowed for consistent prediction of pain probability on a trial-by-trial basis (**Supplemental Figure 2**). Principal Component 1 scores of CS and HP trials were used to train a support vector machine. We chose CS and HP trials because they triggered "non-painful" or "painful" behaviors with high confidence and the corresponding PCA scores showed the most consistent patterns across strain and sex. The trained SVM then predicted the probability of being "pain-like" for other trials. Quite analogously to the PCA based approach, we consistently separated innocuous versus noxious behavioral responses.

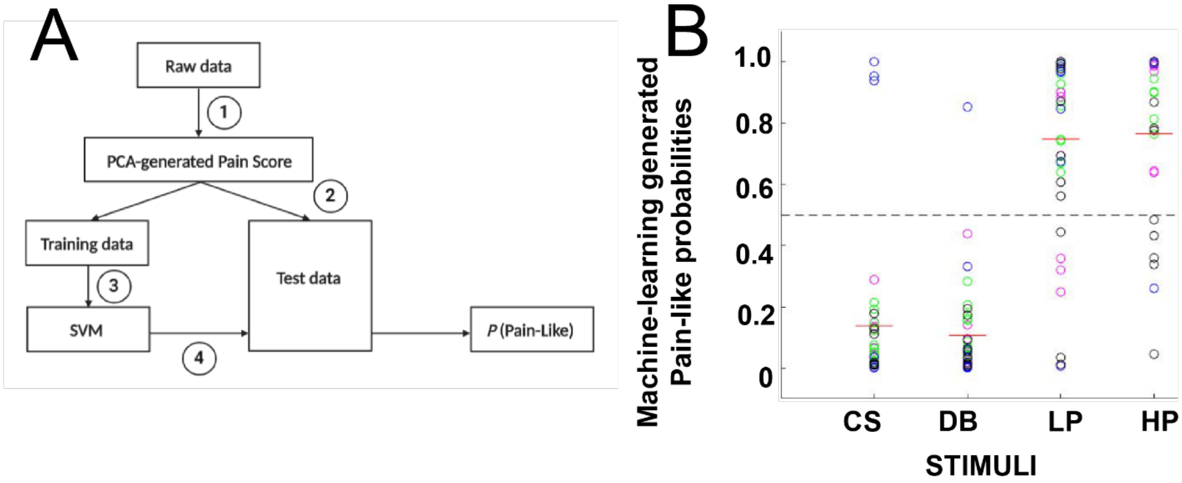

Supplemental Figure 2 . **Trained support vector machine (SVM) predicts the pain-like probabilities for each stimulus.** (A) SVM analytical pipeline. Step (1): calculate PC1 score of each trial by performing PCA on the z-scores from Table 1. Step (2): Split PC1 scores into training and testing sets. Step (3): train SVM with PC1 scores of training data (CS and HP). Step (4): predict pain-like probability (P [pain-like]) of remaining PC scores by training the model with three of the sex + strain combinations and testing on the remaining sex + strain. (B) Machine learning-generated pain-like probabilities of CS, DB, LP, and HP trials in Long Evans and Sprague Dawley female and male rats. Color key: Green = Long Evans Females, Magenta = Long Evans Males, Black = Sprague Dawley Females, Blue = Sprague Dawley Males.

Von Frey hair filaments do not elicit pain-like responses

Applying VFHs stimulation of varying forces resulted in responses that registered in the non-pain domain for nocifensive scores, paw height and velocity.

There were no significant difference in composite nocifensive score across any of the VFHs for male or female SD or LE rats (**Supplemental Figure 3A**; Long Evans Female: $F(1.949, 17.54) = 2.168$, p = 0.1450; Long Evans Male: $F(2.324, 20.92) = 0.5000$, p = 0.6408; Sprague Dawley Female: $F(2.513, 22.61) =0.7663$, p=0.5038; Sprague Dawley Male: $F(2.435, 27.60) = 0.5330$, p=0.6272). When assessing the kinematic movements of paw speed and height, only LE males showed a difference across VFH force for speed (**Supplemental Figure 3B;** Long Evans Female: $F(1.529, 12.23) = 0.6943$, p=0.4807; Long Evans Male: $F(1.905, 20.47) = 8.756$, p=0.0020; Sprague Dawley Female: $F(2.098, 24.48) = 3.029$, p=0.0646; Sprague Dawley Male: $F(2.189, 19.70) = 2.240$, p=0.1294; **Supplemental Figure 3C;** Long Evans Female: $F(2.294,$

25.24) = 2.124, p = 0.1349; Long Evans Male: $F(2.652, 30.06)$ =2.862, p=0.0592; Sprague Dawley Female: $F(1.872, 21.84)$ = 1.690, p=0.2087; Sprague Dawley Male: $F(2.110, 18.99)$ = 3.138, p=0.0642]).

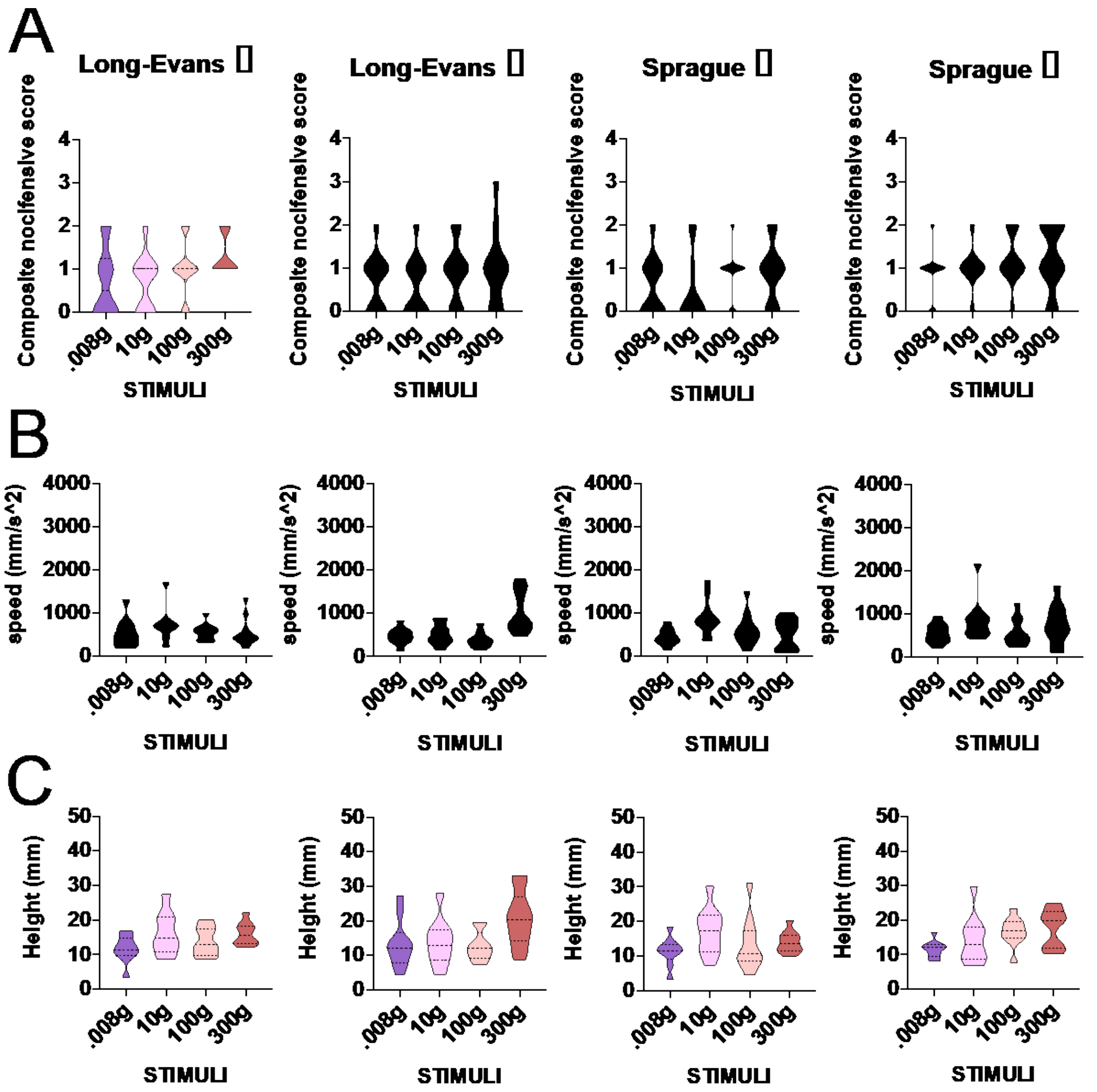

Supplemental Figure 3 . **Sub-second temporal mapping of rat composite nocifensive score and paw kinematic behavioral profile in response to Von Frey hair filaments.** (A) Composite nocifensive score of Long Evans and Sprague Dawley female and male rats (40 rats in total; 10 per group) following stimulation with Von Frey hair filaments of varying forces: 0.008, 10, 100, and 300 g. The score is a composite measurement of eye grimace/orbital tightening, paw shake, jumping, and paw guard. For instance, animals featuring 3 of the 4 behaviors are assigned a score of 3 for that particular trial. (B) Hind-paw kinematic movements evoked by the same VFH filaments. Paw speed of the first paw raise of the stimulated paw is the distance from the initial paw lift to the highest point divided by the time in seconds between the two points. (C) Paw height is the distance from the mesh floor to the highest point following paw stimulation. Truncated violin plot shows the distribution density of the composite scores, with the solid horizontal top and bottom lines representing the minimum and maximum values, respectively; dashed horizontal line for the median value; and dotted top and bottom horizontal lines representing the cutoff for the top 75% and bottom 25% percentile of values, respectively.

Validating the novel pain scale in Long Evans animals. PCA-generated pain scores for male and female Long Evans rats in response to all stimuli were analyzed for each strain. All stimuli tested, including dynamic brush, cotton swab, Von Frey Hairs 0.008g, 10g, 100g, 300g, light pin prick and heavy pin prick were included in the comparison for Long Evans (**Supplemental Figure 4A)** and Sprague Dawley rats. (**Supplemental Figure 4B**). One way ANOVA revealed that the nature of the stimulus applied had a significant impact on the PCA generated pain scores [Long Evans: $F(15,131)=16.02$, $p<.0001$]; Sprague Dawley: [$F(15,131)=6.820$, $p<.0001$]. For Long Evans animals, post-hoc Dunnett tests revealed that only VF300 in males and light and heavy pin pricks in males and females resulted in higher PCA scores than innocuous stimuli (cotton swab). For Sprague Dawley rats, post-hoc Dunnett tests revealed that only light and heavy pin pricks in males and females resulted in higher PCA scores than innocuous stimuli (cotton swab).

A

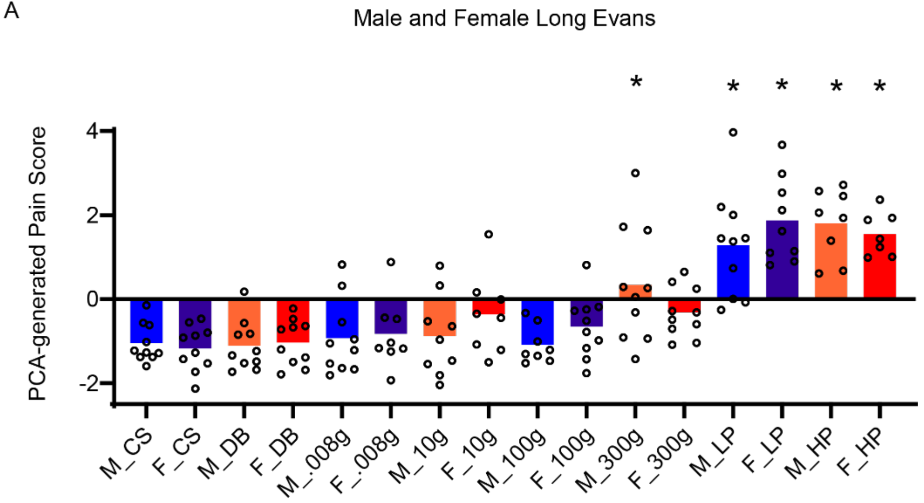

B

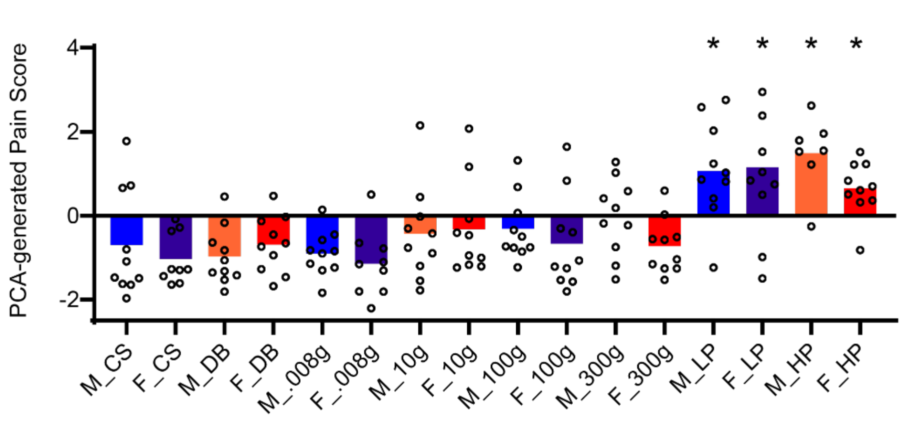

Supplemental Figure 4 . Stimulation with von Frey hair filaments map between innocuous brush stimuli and noxious pinprick using the PCA-generated Pain Score.

(A) PCA-generated pain score for a all stimuli measured: cotton swab (CS), dynamic brush (DB), Von Frey Hairs 0.008g, 10g, 100g, 300g; light pin prick (LP) and heavy pin prick (HP) for males (M) and females (F) Long Evans rats. (B) PCA-generated pain score for a all stimuli measured: cotton swab (CS), dynamic brush (DB), Von Frey Hairs 0.008g, 10g, 100g, 300g; light pin prick (LP) and heavy pin prick (HP) for males (M) and females (F) Sprague Dawley rats. * $p<0.05$ comparing to male CS PCA scores using Dunnett post-hoc tests

Fig. S5. Offspring of morphine-exposed sires show increased sensitivity to morphine-induced antinociception

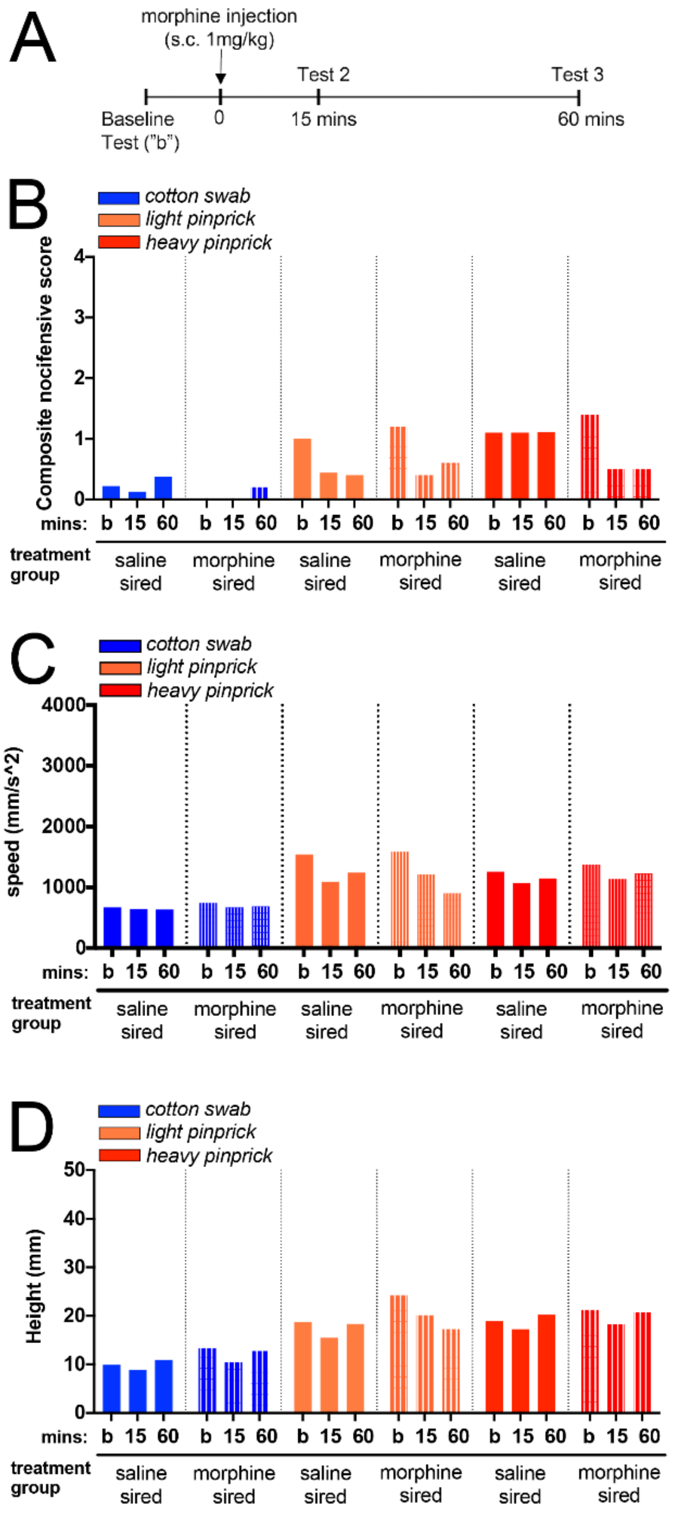

Supplemental Figure 5. **Baseline composite nocifensive score and paw kinematic behavioral profile of first-generation offspring derived from saline-exposed and morphine-exposed sires in response to application of mechanical stimuli to the hind paw** (A) Baseline composite nocifensive score drug-naïve first-generation male derived from sires exposed to wither saline or morphine. The score is a composite measurement of eye grimace/orbital tightening, paw shake, jumping, and paw guard. For instance, animals featuring 3 of the 4 behaviors are assigned a score of 3 for that particular trial. (B) Hind paw kinematic movements evoked by the same VFH filaments. Paw speed of the first paw raise of the stimulated paw is the distance from the initial paw lift to the highest point divided by the time in seconds between the two points. (C) Paw height is the distance from the mesh floor to the highest point following paw stimulation.

Movie S1.

High-speed video showing examples of sub-second nocifensive behaviors that are quantified in the rat pain scale.

Supplemental Table 1. Individual pain score measurements and Z-score transformations, related to Figure 1.

Supplemental Table 2. Individual pain score measurements and Z-score transformations, related to Figure 2.

Supplemental Table 3. Individual pain score measurements and Z-score transformations, related to Figure 4 C-E.

Supplemental Table 4. Individual pain score measurements and Z-score transformations, related to Figure 4 G.

Supplemental Table 5. List of differentially expressed genes in the PAG, comparing male saline-sired to male morphine-sired offspring.

Supplemental Table 6. Enrichment analyses of DEGs in PAG comparing male saline-sired to male morphine-sired progeny.

REFERENCES AND NOTES

1. T. Klengel, B. G. Dias, K. J. Ressler, Models of intergenerational and transgenerational transmission of risk for psychopathology in mice. *Neuropsychopharmacology* **41**, 219–231 (2016).
2. B. G. Dias, K. J. Ressler, Parental olfactory experience influences behavior and neural structure in subsequent generations. *Nat. Neurosci.* **17**, 89–96 (2014).
3. Y. Shin Yim, A. Park, J. Berrios, M. Lafourcade, L. M. Pascual, N. Soares, J. Yeon Kim, S. Kim, H. Kim, A. Waisman, D. R. Littman, I. R. Wickersham, M. T. Harnett, J. R. Huh, G. B. Choi, Reversing behavioural abnormalities in mice exposed to maternal inflammation. *Nature* **549**, 482–487 (2017).
4. R. Kaletsky, R. S. Moore, G. D. Vrla, L. R. Parsons, Z. Gitai, C. T. Murphy, *C. elegans* interprets bacterial non-coding RNAs to learn pathogenic avoidance. *Nature* **586**, 445–451 (2020).
5. H. Szutorisz, J. A. DiNieri, E. Sweet, G. Egervari, M. Michaelides, J. M. Carter, Y. Ren, M. L. Miller, R. D. Blitzer, Y. L. Hurd, Parental THC exposure leads to compulsive heroin-seeking and altered striatal synaptic plasticity in the subsequent generation. *Neuropsychopharmacology* **39**, 1315–1323 (2014).
6. N. L. Yohn, M. S. Bartolomei, J. A. Blendy, Multigenerational and transgenerational inheritance of drug exposure: The effects of alcohol, opiates, cocaine, marijuana, and nicotine. *Prog. Biophys. Mol. Biol.* **118**, 21–33 (2015).
7. L. R. Goldberg, T. J. Gould, Multigenerational and transgenerational effects of paternal exposure to drugs of abuse on behavioral and neural function. *Eur. J. Neurosci.* **50**, 2453–2466 (2019).
8. T. L. Bale, Epigenetic and transgenerational reprogramming of brain development. *Nat. Rev. Neurosci.* **16**, 332–344 (2015).
9. B. R. Carone, L. Fauquier, N. Habib, J. M. Shea, C. E. Hart, R. Li, C. Bock, C. Li, H. Gu, P. D. Zamore, A. Meissner, Z. Weng, H. A. Hofmann, N. Friedman, O. J. Rando, Paternally induced transgenerational environmental reprogramming of metabolic gene expression in mammals. *Cell* **143**, 1084–1096 (2010).
10. F. M. Vassoler, A. M. Toorie, D. N. Teceno, P. Walia, D. J. Moore, T. D. Patton, E. M. Byrnes, Paternal morphine exposure induces bidirectional effects on cocaine versus opioid self-administration. *Neuropharmacology* **162**, 107852 (2020).

11. M. Z. Farah Naquiah, R. J. James, S. Suratman, L. S. Lee, M. I. Mohd Hafidz, M. Z. Salleh, L. K. Teh, Transgenerational effects of paternal heroin addiction on anxiety and aggression behavior in male offspring. *Behav. Brain Funct.* **12**, 23 (2016).
12. A. S. Ellis, A. B. Toussaint, M. C. Knouse, A. S. Thomas, A. R. Bongiovanni, H. L. Mayberry, S. Bhakta, K. Peer, D. A. Bangasser, M. E. Wimmer, Paternal morphine self-administration produces object recognition memory deficits in female, but not male offspring. *Psychopharmacology*, **237**, 1209–1221 (2020).
13. G. Ashabi, M. S. Sadat-Shirazi, A. Akbarabadi, N. Vousooghi, Z. Kheiri, H. Toolee, S. Khalifeh, M. R. Zarrindast, Is the nociception mechanism altered in offspring of morphine-abstinent rats? *J. Pain* **19**, 529–541 (2018).
14. T. J. Cicero, B. Nock, L. O'Connor, M. Adams, E. R. Meyer, Adverse effects of paternal opiate exposure on offspring development and sensitivity to morphine-induced analgesia. *J. Pharmacol. Exp. Ther.* **273**, 386–392 (1995).
15. D. Le Bars, M. Gozariu, S. W. Cadden, Animal models of nociception. *Pharmacol. Rev.* **53**, 597–652 (2001).
16. J. S. Mogil, Animal models of pain: Progress and challenges. *Nat. Rev. Neurosci.* **10**, 283–294 (2009).
17. S. Bourane, K. S. Grossmann, O. Britz, A. Dalet, M. G. Del Barrio, F. J. Stam, L. Garcia-Campmany, S. Koch, M. Goulding, Identification of a spinal circuit for light touch and fine motor control. *Cell* **160**, 503–515 (2015).
18. S. S. Ranade, S. H. Woo, A. E. Dubin, R. A. Moshourab, C. Wetzel, M. Petrus, J. Mathur, V. Begay, B. Coste, J. Mainquist, A. J. Wilson, A. G. Francisco, K. Reddy, Z. Z. Qiu, J. N. Wood, G. R. Lewin, A. Patapoutian, Piezo2 is the major transducer of mechanical forces for touch sensation in mice. *Nature* **516**, 121–125 (2014).
19. B. Duan, L. Cheng, S. Bourane, O. Britz, C. Padilla, L. Garcia-Campmany, M. Krashes, W. Knowlton, T. Velasquez, X. Ren, S. E. Ross, B. B. Lowell, Y. Wang, M. Goulding, Q. Ma, Identification of spinal circuits transmitting and gating mechanical pain. *Cell* **159**, 1417–1432 (2014).
20. S. E. Murthy, M. C. Loud, I. Daou, K. L. Marshall, F. Schwaller, J. Kuhnemund, A. G. Francisco, W. T. Keenan, A. E. Dubin, G. R. Lewin, A. Patapoutian, The mechanosensitive ion channel Piezo2 mediates sensitivity to mechanical pain in mice. *Sci. Transl. Med.* **10**, eaat9897 (2018).

21. N. T. Fried, A. Chamessian, M. J. Zylka, I. Abdus-Saboor, Improving pain assessment in mice and rats with advanced videography and computational approaches. *Pain* **161**, 1420–1424 (2020).
22. I. Abdus-Saboor, N. T. Fried, M. Lay, J. Burdge, K. Swanson, R. Fischer, J. Jones, P. Dong, W. Cai, X. Guo, Y. X. Tao, J. Bethea, M. Ma, X. Dong, L. Ding, W. Luo, Development of a mouse pain scale using sub-second behavioral mapping and statistical modeling. *Cell Rep.* **28**, 1623–1634.e4 (2019).
23. J. M. Jones, W. Foster, C. R. Twomey, J. Burdge, O. M. Ahmed, T. D. Pereira, J. A. Wojick, G. Corder, J. B. Plotkin, I. Abdus-Saboor, A machine-vision approach for automated pain measurement at millisecond timescales. *eLife* **9**, e57258 (2020).
24. D. J. Langford, A. L. Bailey, M. L. Chanda, S. E. Clarke, T. E. Drummond, S. Echols, S. Glick, J. Ingrao, T. Klassen-Ross, M. L. Lacroix-Fralish, L. Matsumiya, R. E. Sorge, S. G. Sotocinal, J. M. Tabaka, D. Wong, A. M. van den Maagdenberg, M. D. Ferrari, K. D. Craig, J. S. Mogil, Coding of facial expressions of pain in the laboratory mouse. *Nat. Methods* **7**, 447–449 (2010).
25. A. Francois, N. Schuetter, S. Laffray, J. Sanguesa, A. Pizzoccaro, S. Dubel, A. Mantilleri, J. Nargeot, J. Noel, J. N. Wood, A. Moqrich, O. Pongs, E. Bourinet, The low-threshold calcium channel Cav3.2 determines low-threshold mechanoreceptor function. *Cell Rep.* **10**, 370–382 (2015).
26. S. H. Woo, S. Ranade, A. D. Weyer, A. E. Dubin, Y. Baba, Z. Qiu, M. Petrus, T. Miyamoto, K. Reddy, E. A. Lumpkin, C. L. Stucky, A. Patapoutian, Piezo2 is required for Merkel-cell mechanotransduction. *Nature* **509**, 622–626 (2014).
27. J. Mika, M. Osikowicz, W. Makuch, B. Przewlocka, Minocycline and pentoxifylline attenuate allodynia and hyperalgesia and potentiate the effects of morphine in rat and mouse models of neuropathic pain. *Eur. J. Pharmacol.* **560**, 142–149 (2007).
28. D. G. Williams, A. Dickenson, M. Fitzgerald, R. F. Howard, Developmental regulation of codeine analgesia in the rat. *Anesthesiology* **100**, 92–97 (2004).
29. K. Popiolek-Barczyk, D. Lazewska, G. Latacz, A. Olejarz, W. Makuch, H. Stark, K. Kiec-Kononowicz, J. Mika, Antinociceptive effects of novel histamine H3 and H4 receptor antagonists and their influence on morphine analgesia of neuropathic pain in the mouse. *Br. J. Pharmacol.* **175**, 2897–2910 (2018).
30. H. L. Bu, Y. Z. Xia, P. M. Liu, H. M. Guo, C. Yuan, X. C. Fan, C. Huang, Y. Y. Wen, C. L. Kong, T. Wang, L. T. Ma, X. X. Li, H. W. Zhang, L. R. Zhang, M. Y. Ma, Y. Q. Ai, W. Zhang, The roles

of chemokine CXCL13 in the development of bone cancer pain and the regulation of morphine analgesia in rats. *Neuroscience* **406**, 62–72 (2019).

31. M. Barrot, Tests and models of nociception and pain in rodents. *Neuroscience* **211**, 39–50 (2012).
32. R. Schmidt, M. Schmelz, M. Ringkamp, H. O. Handwerker, H. E. Torebjork, Innervation territories of mechanically activated C nociceptor units in human skin. *J. Neurophysiol.* **78**, 2641–2648 (1997).
33. I. Kissin, P. T. Brown, E. L. Bradley Jr., Sedative and hypnotic midazolam-morphine interactions in rats. *Anesth. Analg.* **71**, 137–143 (1990).
34. F. V. Abbott, E. R. Guy, Effects of morphine, pentobarbital and amphetamine on formalin-induced behaviours in infant rats: Sedation versus specific suppression of pain. *Pain* **62**, 303–312 (1995).
35. R. Fog, Behavioural effects in rats of morphine and amphetamine and of a combination of the two drugs. *Psychopharmacologia* **16**, 305–312 (1970).
36. S. Toyama, N. Shimoyama, Y. Tagaito, H. Nagase, T. Saitoh, M. Yanagisawa, M. Shimoyama, Nonpeptide orexin-2 receptor agonist attenuates morphine-induced sedative effects in rats. *Anesthesiology* **128**, 992–1003 (2018).
37. F. M. Vassoler, M. E. Wimmer, Consequences of parental opioid exposure on neurophysiology, behavior, and health in the next generations. *Cold Spring Harb. Perspect. Med.* **11**, a040436 (2021).
38. P. S. Eriksson, L. Ronnback, E. Hansson, Do persistent morphine effects involve interactions with the genome? *Drug Alcohol Depend.* **24**, 39–43 (1989).
39. N. Pachenari, H. Azizi, E. Ghasemi, M. Azadi, S. Semnanian, Exposure to opiates in male adolescent rats alters pain perception in the male offspring. *Behav. Pharmacol.* **29**, 255–260 (2018).
40. C. B. Pert, S. H. Snyder, Opiate receptor: Demonstration in nervous tissue. *Science* **179**, 1011–1014 (1973).
41. H. Sims-Williams, J. C. Matthews, P. S. Talbot, S. Love-Jones, J. C. Brooks, N. K. Patel, A. E. Pickering, Deep brain stimulation of the periaqueductal gray releases endogenous opioids in humans. *NeuroImage* **146**, 833–842 (2017).
42. C. Li, J. A. Sugam, E. G. Lowery-Gionta, Z. A. McElligott, N. M. McCall, A. J. Lopez, J. M. McKlveen, K. E. Pleil, T. L. Kash, Mu opioid receptor modulation of dopamine neurons in the periaqueductal gray/dorsal raphe: A role in regulation of pain. *Neuropsychopharmacology* **41**, 2122–2132 (2016).
43. D. J. Mayer, T. L. Wolfle, H. Akil, B. Carder, J. C. Liebeskind, Analgesia from electrical stimulation in the brainstem of the rat. *Science* **174**, 1351–1354 (1971).

44. S. Hollinger, J. R. Hepler, Cellular regulation of RGS proteins: Modulators and integrators of G protein signaling. *Pharmacol. Rev.* **54**, 527–559 (2002).
45. N. B. Senese, R. Kandasamy, K. E. Kochan, J. R. Traynor, Regulator of G-protein signaling (RGS) protein modulation of opioid receptor signaling as a potential target for pain management. *Front. Mol. Neurosci.* **13**, 5 (2020).
46. J. T. Lamberts, E. M. Jutkiewicz, R. M. Mortensen, J. R. Traynor, mu-Opioid receptor coupling to Gα(o) plays an important role in opioid antinociception. *Neuropsychopharmacology* **36**, 2041–2053 (2011).
47. J. T. Lamberts, J. R. Traynor, Opioid receptor interacting proteins and the control of opioid signaling. *Curr. Pharm. Des.* **19**, 7333–7347 (2013).
48. E. O. Sanchez, C. C. Bavley, A. U. Deutschmann, R. Carpenter, D. R. Peterson, R. Karbalaei, J. Flowers II, C. M. Rogers, M. G. Langrehr, C. S. Ardekani, S. T. Famularo, A. R. Bongiovanni, M. C. Knouse, S. B. Floresco, L. A. Briand, M. E. Wimmer, D. A. Bangasser, Early life adversity promotes resilience to opioid addiction-related phenotypes in male rats and sex-specific transcriptional changes. *Proc. Natl. Acad. Sci.* **118**, e2020173118 (2021).
49. S. M. E. Sahraeian, M. Mohiyuddin, R. Sebra, H. Tilgner, P. T. Afshar, K. F. Au, N. B. Asadi, M. B. Gerstein, W. H. Wong, M. P. Snyder, E. Schadt, H. Y. K. Lam, Gaining comprehensive biological insight into the transcriptome by performing a broad-spectrum RNA-seq analysis. *Nat. Commun.* **8**, 59 (2017).
50. L. Cui, X. Miao, L. Liang, I. Abdus-Saboor, W. Olson, M. S. Fleming, M. Ma, Y. X. Tao, W. Luo, Identification of early RET+ deep dorsal spinal cord interneurons in gating pain. *Neuron* **91**, 1137–1153 (2016).
51. S. Andrews, *FastQC: a quality control tool for high throughput sequence data* (Babraham Bioinformatics, Babraham Institute, Cambridge, United Kingdom, 2010).
52. A. M. Bolger, M. Lohse, B. J. B. Usadel, Trimmomatic: A flexible trimmer for Illumina sequence data. *Bioinformatics* **30**, 2114–2120 (2014).
53. J. Sirén, N. Välimäki, V. Mäkinen, HISAT2-fast and sensitive alignment against general human population. *IEEE/ACM Trans. Comput. Biol. Bioninforma.* **11**, 375–388 (2014).
54. M. Pertea, G. M. Pertea, C. M. Antonescu, T.-C. Chang, J. T. Mendell, S. L. J. N. b. Salzberg, StringTie enables improved reconstruction of a transcriptome from RNA-seq reads. *Nat. Biotechnol.* **33**, 290 (2015).

55. M. I. Love, W. Huber, S. Anders, Moderated estimation of fold change and dispersion for RNA-seq data with DESeq2. *Genome Biol.* **15**, 550 (2014).
56. R Core Team, *R: A Language and Environment for Statistical Computing* (R Foundation for Statistical Computing, 2013).
57. S. Heinz, C. Benner, N. Spann, E. Bertolino, Y. C. Lin, P. Laslo, J. X. Cheng, C. Murre, H. Singh, C. K. Glass, Simple combinations of lineage-determining transcription factors prime cis-regulatory elements required for macrophage and B cell identities. **38**, 576–589 (2010).
58. G. Yu, Q.-Y. He, ReactomePA: An R/Bioconductor package for reactome pathway analysis and visualization. *Mol. BioSyst.* **12**, 477–479 (2016).
59. S. B. Plaisier, R. Taschereau, J. A. Wong, T. G. Graeber, Rank–rank hypergeometric overlap: Identification of statistically significant overlap between gene-expression signatures. *Nucleic Acids Res.* **38**, e169 (2010).
60. K. M. Cahill, Z. Huo, G. C. Tseng, R. W. Logan, M. L. Seney, Improved identification of concordant and discordant gene expression signatures using an updated rank-rank hypergeometric overlap approach. **8**, 9588 (2018).
